# Genetic Variation in Fruit-to-Grain Conversion Efficiency in *Coffea canephora*: Heritability, Temporal Instability, and Divergence in Robusta Hybrids and Conilon

**DOI:** 10.3390/biology15120899

**Published:** 2026-06-08

**Authors:** Deurimar Herênio Gonçalves Júnior, Jéssica Almeida Jorge, Júlio César Pereira Machado, Danillo Lima Pereira, Weverton Pereira Rodrigues, Fábio Luiz Partelli

**Affiliations:** 1 Departamento de Ciências Agrárias e Biológicas, North University Center of Espírito Santo (CEUNES), Federal University of Espírito Santo (UFES), São Mateus 29932-900, Espírito Santo, Brazil; jessicaalmeidajorge2@gmail.com (J.A.J.); danillo.pereira@edu.ufes.br (D.L.P.); 2 Centro de Ciências Agrárias, Universidade Estadual da Região Tocantina do Maranhão, Imperatriz 65901-480, Maranhão, Brazil

**Keywords:** *Coffea canephora*, Bayesian heritability, genotype × environment interaction, temporal stability, indirect selection, genetic divergence

## Abstract

The efficiency of converting ripe fruits into processed beans is an economically relevant component of *Coffea canephora* production systems, yet its genetic parameters remain poorly characterized in studies that do not partition the genotype × year (G×Y) interaction. This study estimated genetic parameters for five processing efficiency traits, namely grain proportion (% grain), husk proportion (% husk), fruit fresh mass per grain mass (FWM/GW), fruit fresh mass per bag (FWM/bag), and fruit volume per bag (FVol/bag), in 48 *C. canephora* genotypes (40 Robusta, 8 Conilon) evaluated over two crop years (2023–2024) in Jaguaré, Espírito Santo, Brazil. Bayesian inference via MCMC (brms) revealed that the genotype × year variance component exceeded the genotypic variance in 79–97% of posterior samples across the 48 genotypes evaluated over two crop years, a result that should be interpreted within the context of this restricted temporal window, with median heritabilities of 0.27–0.50 (95% credible intervals spanning up to 0.66 units, reflecting the uncertainty inherent to the two-year evaluation window) and genotypic correlations of 0.19–0.38 between years, indicating low consistency of genetic merit across crop seasons. Bayesian probability of consistent superiority identified Z21 as the genotype with the highest predictability for FWM/bag (prob. =0.846 at 20% selection intensity), while VR3 showed a favorable profile across four traits simultaneously. The multi-trait model with unstructured covariance estimated a negative genetic correlation between % grain and FWM/bag (${\hat{r}}_{g}=-0.87$), suggesting potential for indirect selection. UPGMA clustering based on Mahalanobis distance (CCC =0.813) yielded six divergence groups that did not coincide with the botanical classification Conilon/Robusta. In this single-location, two-year study, temporal instability was the predominant source of uncertainty in the selection for processing efficiency in *C. canephora* under restricted evaluation windows. under restricted evaluation windows. Accordingly, the highlighted genotypes should be interpreted as priority candidates for validation in multi-environment, multi-year networks, rather than as definitive cultivar recommendations, given that the short evaluation window limits the generalizability of genotypic rankings.

## Simple Summary

Coffee farming profitability depends not only on yield per area but also on how efficiently harvested fruits are converted into processed beans. A variety that requires less fruit to fill a commercial bag reduces harvesting and processing costs, yet this trait is rarely prioritized in breeding programs. This study evaluated 48 varieties of *Coffea canephora*, the species behind Robusta and Conilon coffees, over two growing seasons in northern Espírito Santo, Brazil. Using Bayesian statistical methods, we estimated how much of the variation in processing efficiency is genetic and how much depends on the growing conditions of each year. We found that variety performance changed considerably between years, meaning that selecting varieties based on a single season can be misleading. Despite this instability, two varieties, Z21 and VR3, consistently showed superior processing efficiency across both seasons and are promising candidates for broader trials. We also found that a simpler measurement, the proportion of grain in the fruit, can predict which varieties will need less fruit per processed bag, potentially simplifying large-scale variety screening. These results highlight the importance of multi-year evaluations in coffee breeding and offer practical guidance for improving processing efficiency in Conilon and Robusta production systems.

## 1. Introduction

Coffee cultivation, particularly *Coffea canephora*, occupies a strategic position in Brazilian coffee production, with growing relevance in intensive farming systems across the Northern, Northeastern, and Southeastern regions of the country. Genetic improvement and agronomic management of *Coffea canephora* have historically prioritized yield per area and adaptation to environmental stresses, with recent advances consolidating these gains through genomic tools and multi-environment evaluation frameworks [1]. However, traits related to the conversion efficiency of harvested fruit into processed beans, directly tied to industrial yield and operational costs, remain comparatively underexplored, despite their practical importance for the profitability of production systems. This gap is particularly relevant because agronomic productivity, expressed as fruit volume or mass per area, does not always translate proportionally into greater quantities of processed coffee. Genotypes with similar productivity may thus differ substantially in the fruit mass required to produce a commercial bag, affecting the costs of harvesting, drying, transport, processing, and storage.

The efficiency of converting ripe fruits into processed beans is a component of the *C. canephora* production system that rarely figures as a primary selection criterion in breeding programs, yet its economic implications for growers are direct and measurable. The fruit fresh mass required to obtain a 60 kg bag of processed coffee, here designated FWM/bag, varies considerably among genotypes and determines, in combination with yield per area, the operational cost of harvesting and post-harvest processing per unit of marketable product [1,2]. In clonal production systems of *C. canephora* in Espírito Santo and Rondônia, where manual strip harvesting accounts for a significant share of production costs [3], differences of only a few kilograms per bag between high- and low-efficiency genotypes can represent economically meaningful variation at the scale of a commercial harvest.

Genetic variation in grain proportion and gravimetric processing efficiency in *C. canephora* has been documented in studies conducted predominantly in Espírito Santo [2,4,5], with broad-sense heritability estimates frequently exceeding 0.60 for % grain and 0.70 for physical yield traits. These estimates, however, derive mostly from single-year experiments or from analyses that do not explicitly partition the genotype × year interaction (G×Y) as an independent variance component. In models lacking G×Y, the differential environmental sensitivity of genotypes may remain confounded with the genotypic component, potentially inflating heritability estimates and overstating the predictability of selection relative to what the data support [6,7]. The primary gap, therefore, lies not merely in the scarcity of studies on physical yield in *C. canephora*, but in the need to estimate these traits under models capable of separating permanent genetic merit from the genotype-specific response to different crop years, a distinction critical for breeding programs, since rankings obtained in a single cycle may reflect circumstantial performance rather than stable genetic superiority.

For perennial crops such as *C. canephora*, this limitation is compounded by yield biennialism and the physiological carry-over between successive harvests, which can introduce temporal structure into phenotypic variation and hinder the separation of year effects, G×Y interaction, and residual variance [8]. Multi-environment studies with the species have shown that G×E interaction can be substantial even for traits with moderate to high heritability [4,5,9], yet the probabilistic quantification of temporal instability, expressed here as posterior probabilities of G×Y dominance and of consistent genotypic superiority across years, remains poorly explored in the breeding literature for processing efficiency traits in the species (though interannual instability has been documented for yield and adaptability traits in *C. canephora* [4,5]. The distinction matters: a simple estimate of $\sigma_{G\times Y}^{2}$ is less informative to the breeder than knowing, for each candidate genotype, the probability of consistent performance across years under varying selection intensities.

The genetic correlation among processing efficiency traits in *C. canephora* is also poorly documented. Knowing whether selection for % grain, operationally simpler as it does not require weighing processed lots, produces a correlated response in FWM/bag has direct implications for the feasibility of indirect selection strategies in large panels. Multi-trait models with unstructured covariance allow these correlations to be estimated by variance component, genetic, interaction, and residual factors, distinguishing associations attributable to the genetic component from those driven by interaction and residual components [10,11]. Based on evidence from perennial crops showing that physical yield traits are particularly sensitive to interannual environmental variation [8,12], we hypothesized that the genotype × year interaction variance would exceed the genotypic variance for processing efficiency traits in *C. canephora*, thereby limiting the reliability of single-cycle selection.

This study evaluated 48 *C. canephora* genotypes (40 Robusta hybrids and eight Conilon) over two harvest years (2023–2024) in Jaguaré, northern Espírito Santo, Brazil, with the objectives of estimating genetic parameters for five processing efficiency traits by full Bayesian inference, decomposing variance into genotypic, G×Y, and residual components, quantifying the probability of consistent superiority across years for each genotype, estimating genetic correlations among traits using a multi-trait model with unstructured covariance, and characterizing the genetic divergence of the panel. The results are interpreted in the context of the opportunities and limitations of selection for processing efficiency in *C. canephora* breeding programs with restricted temporal evaluation windows.

## 2. Materials and Methods

### 2.1. Experimental Site and Edaphoclimatic Conditions

The experiment was conducted in a commercial coffee plantation in the municipality of Jaguaré, northern Espírito Santo, Brazil ($18{}^{\circ}59^{\prime }33^{\prime \prime }$ S; $39{}^{\circ}55^{\prime }07^{\prime \prime }$ W; approximate altitude of 50 m). The regional climate is classified as Aw according to the Köppen-Geiger system [13,14], with a rainy season concentrated between October and March and a dry period between April and September. The soil is classified as a Dystrophic Yellow Latosol [15], correlated to a Xanthic Dystric Ferralsol under the World Reference Base for Soil Resources [16].

**Figure 1:**
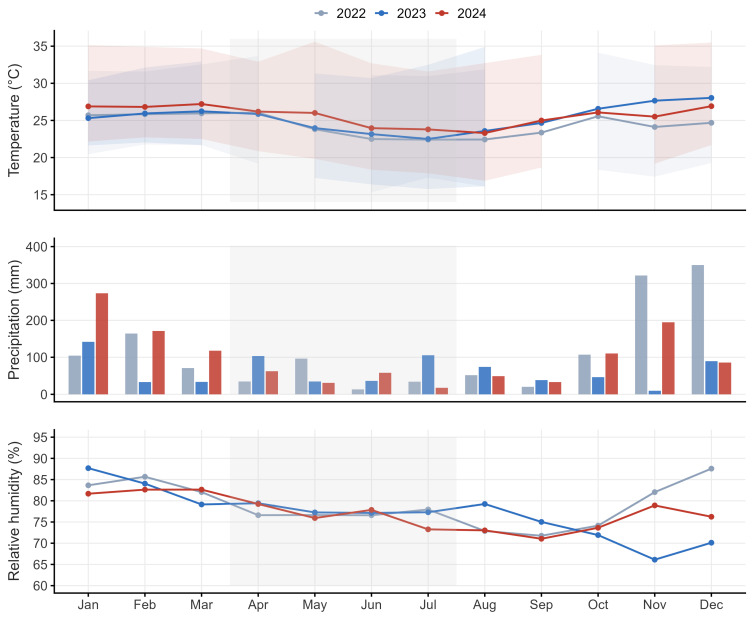
Climatic conditions in the municipality of Jaguaré, Espírito Santo, during the experimental period (2022–2024), retrieved from NASA POWER (0.5° × 0.5° resolution; lat. −18.99°; lon. −40.08°). From top to bottom: monthly mean temperature (°C, line) and daily thermal amplitude (shaded band); monthly cumulative precipitation (mm); and monthly mean relative humidity (%). The grey rectangle indicates the harvest period (April–July), coinciding with fruit filling and maturation. Colors identify years: 2022 (grey), 2023 (blue), and 2024 (red).

Climatic data were obtained from NASA POWER [17], as the experimental site is located in a commercial farm without an on-site meteorological station, and no public or institutional station was available within a representative distance of the experimental area. Although the 0.5° × 0.5° resolution ( 55 km) may not capture fine-scale microclimatic variation, this data source has been widely adopted in coffee agronomy studies conducted under similar conditions [5,9]. The substantial interannual differences in precipitation (747 mm in 2023 vs. 1206 mm in 2024) and temperature documented from this source (Figure 1) provide direct evidence of environmental contrast between the two evaluated years, supporting the interpretation of the genotype × year interaction as a response to genuinely distinct growing conditions.

### 2.2. Plant Material and Experimental Design

A total of 48 *Coffea canephora* Pierre ex Froehner genotypes were evaluated, 40 belonging to the Robusta group and eight to the Conilon group (Table 1). The genotypes were selected to represent the main genetic groups cultivated in the northern Espírito Santo and Rondônia production regions, comprising elite materials from breeding programs conducted by Embrapa Café and Incaper, as well as accessions from germplasm collections with documented agronomic potential. The panel was not a random sample from a broader population but rather a purposive selection aimed at capturing the relevant genetic diversity available for commercial cultivation in the region. Among the Robusta genotypes, 39 originated from the state of Rondônia, sourced from selection programs conducted by the Brazilian Agricultural Research Corporation (Embrapa Café) and the Capixaba Institute for Research, Technical Assistance and Rural Extension (Incaper), while one genotype (A1), originally classified as Conilon from Espírito Santo, was recently reclassified as an interspecific hybrid by genomic analysis [18]. The eight Conilon genotypes originated from Espírito Santo and Bahia, belonging to germplasm collections described in [5].

Planting was carried out in April 2021 at a spacing of 3.0 m between rows and 0.6 m between plants, resulting in a plant density of 5556 plants ha^−1^. Plants were trained to a two-orthotropic-stem system, consistent with commercial management practices adopted for the species in the northern Espírito Santo production region [19]. The experimental design was a randomized complete block design with 48 treatments and three replications. Each plot consisted of seven plants, of which the five central plants were considered the effective experimental unit to minimize border effects.

### 2.3. Experimental Management

The experiment was conducted under drip irrigation, adjusted to the phenological stage of the crop to prevent water deficit during flowering, fruit filling, and maturation. Although irrigation was managed to reduce the occurrence of severe water deficit, interannual differences in precipitation, temperature, and atmospheric demand were retained as relevant environmental components, given that supplemental irrigation does not fully eliminate the effects of climate on fruit growth, maturation, and physical composition. Fertilization followed the technical recommendations for *C. canephora* in Espírito Santo [20,21], with nutrient splitting and sources compatible with commercial production systems for the species in the region. Phytosanitary control included preventive applications for the main pests and diseases of *C. canephora* in the region, notably leaf miner (*Leucoptera coffeella*) and coffee leaf rust (*Hemileia vastatrix*), following the integrated pest management recommendations adopted for commercial plantations in northern Espírito Santo [19] Weed management was carried out by mechanical mowing between rows and manual weeding within rows, applied uniformly across all plots throughout the experimental period.

### 2.4. Sample Collection and Evaluated Traits

Harvesting was conducted in a staggered manner between April and July of 2023 and 2024, following the individual maturation stage of each genotype. June corresponded to the period of highest harvest concentration, coinciding with peak maturation in the northern Espírito Santo region, but genotypes with earlier or later maturation cycles were harvested accordingly within the April-to-July window. For each plot, harvest was performed when approximately 80% of fruits had reached the cherry stage, by selective manual strip picking. Three independent samples of 25 ripe fruits per plot were collected from branches distributed across different canopy strata of the plant, totaling 75 fruits per genotype per block. Samples were immediately transported to the laboratory for processing.

Fruits were weighed on a precision analytical balance (0.001 g) to determine fresh mass. Volume was determined by the water displacement method using a 100 mL graduated cylinder filled with distilled water, with the difference between initial and final volumes corresponding to the total sample volume. Samples were then dried in a forced-air oven at 50 °C until constant mass was reached, a criterion verified when the variation between two consecutive weighings at 24-h intervals was less than 0.5%. After complete drying, manual dehulling was performed to separate the grain fraction (dry endosperm) from the husk fraction (pericarp, mesocarp, and endocarp), with each fraction weighed independently.

From these measurements, six yield traits were calculated. Grain proportion and husk proportion were expressed as the percentage of each component relative to the total dry fruit mass. The ratio of ripe fruit fresh mass to processed grain mass (FWM/GW) was obtained from the ratio of fruit fresh mass to dry grain mass, corrected to 12% moisture content, as shown in the following equation:

(1) $$\mathrm{FWM}/\mathrm{GW}=\frac{M_{\mathrm{fruit}}}{M_{\mathrm{grain}}\times \left(1-0.12\right)}$$

where $M_{\mathrm{fruit}}$ is the fresh mass of ripe fruits (g) and $M_{\mathrm{grain}}$ is the dry mass of the grain fraction (g), with the factor (1−0.12) correcting grain mass to a standardized 12% moisture content basis. The 12% moisture content was adopted as the standard commercial reference for processed coffee in Brazil [19], applied uniformly to all samples. Grain samples were dried in a forced-air oven at 50 °C until constant mass prior to weighing, as described above, ensuring that $M_{\mathrm{grain}}$ represents the dry mass basis from which the commercial moisture correction was applied. The ripe fruit fresh mass required to obtain a 60 kg bag of processed coffee (FWM/bag) and the equivalent volume (FVol/bag) were derived directly from FWM/GW, using the commercial reference of 60 kg of processed beans at 12% moisture content. The ratio of fruit volume to fruit fresh mass (FVol/FWM) was calculated as an indicator of apparent fruit density, expressed in mL g^−1^.

### 2.5. Statistical Analysis

#### 2.5.1. Mixed Model Selection

The mixed model structure was defined through sequential steps prior to genetic parameter estimation. The inclusion of a permanent plot effect (temporal split-plot) was first tested to account for the dependence between repeated measurements on the same individuals across the two years. Model comparison with and without this component was performed using the Watanabe–Akaike Information Criterion (WAIC) and leave-one-out cross-validation (LOO), implemented in the loo package [22]. For all evaluated traits, the difference in ELPD (expected log pointwise predictive density) between models was less than two standard errors (elpd_diff <2× se_diff), indicating predictive equivalence and rendering the permanent plot effect unnecessary. This conclusion was further supported by likelihood ratio tests (LRT) under the frequentist framework: the permanent plot effect was non-significant for all traits (p=1.00 for % grain, % husk, FVol/bag, and FVol/FWM; p=0.57 for FWM/GW and FWM/bag), with ΔAIC = −2 in all cases, confirming that its inclusion did not improve model fit. It should be noted, however, that with only two years of evaluation, the power to detect a permanent plot component is inherently limited, and the conclusion of predictive equivalence should be interpreted within this constraint. The year × block interaction was tested by model comparison via the Akaike Information Criterion (AIC), revealing no significant effect for any of the evaluated traits (ΔAIC > 0 in all cases; Figure S3), and the term was excluded from the final model. The adopted model was:

(2) $$y_{ijk}=\mu +\alpha_{j}+\beta_{k}+g_{i}+{\left(g\alpha \right)}_{ij}+\varepsilon_{ijk}$$

where $y_{ijk}$ is the observed value of genotype *i* in block *k* and year *j*; μ is the overall mean; $\alpha_{j}$ is the fixed effect of year (j=2023,2024); $\beta_{k}$ is the fixed effect of block (k=1,2,3); $g_{i}$ is the random effect of genotype (i=1,…,48), with $g_{i}∼N\left(0,\sigma_{g}^{2}\right)$; ${\left(g\alpha \right)}_{ij}$ is the random effect of the genotype × year interaction, with ${\left(g\alpha \right)}_{ij}∼N\left(0,\sigma_{gy}^{2}\right)$; and $\varepsilon_{ijk}$ is the residual error, with $\varepsilon_{ijk}∼N\left(0,\sigma_{e}^{2}\right)$. Block was treated as a fixed effect because the three blocks were deliberately assigned spatial positions within the experimental area and are not considered a random sample from a broader population of possible blocks. This specification is standard in randomized complete block designs where blocks represent specific environmental gradients within a single location [6].

The normality of model residuals was assessed visually through posterior predictive checks implemented in brms, comparing the observed data distribution against distributions simulated from the fitted model. No substantial departures from normality were detected for any of the evaluated traits.

#### 2.5.2. Bayesian Inference and Genetic Parameter Estimation

Genetic parameters were estimated by Bayesian inference via Markov chain Monte Carlo (MCMC), implemented in the brms package [23], which uses the HMC-NUTS sampler from Stan [24]. For each trait, four independent chains were run with 4000 iterations each (2000 warmup), yielding 8000 posterior samples in total. Prior distributions of σ∼Student-t(3,0,2.5) were adopted for all standard deviation parameters, and Normal(0,5) for regression coefficients. The Student-*t*(3, 0, 2.5) prior provides moderate regularization with heavier tails than a Half-Normal, which is particularly suitable for variance components that may be near zero, as it assigns non-negligible probability mass to small values without forcing shrinkage toward zero [25]. Convergence was assessed by the $\hat{R}$ diagnostic for all parameters and by the effective number of samples $n_{\mathrm{eff}}>400$. No post-warmup divergences were detected in any of the fitted models.

Mean-basis heritability ($H^{2}$) was estimated from posterior distribution samples as:

(3) $$H^{2}=\frac{\sigma_{g}^{2}}{\sigma_{g}^{2}+\frac{\sigma_{gy}^{2}}{n_{y}}+\frac{\sigma_{e}^{2}}{n_{y}\cdot n_{r}}}$$

where $n_{y}$ is the number of years and $n_{r}$ is the number of replications. The genotypic correlation between years ($r_{g}$) was derived from posterior samples of $\sigma_{g}^{2}$ and $\sigma_{gy}^{2}$:

(4) $$r_{g}=\frac{\sigma_{g}^{2}}{\sigma_{g}^{2}+\sigma_{gy}^{2}}$$

Ninety-five percent credible intervals (95% CI) were obtained directly from the marginal posterior distributions of each parameter.

The heritability estimated in this study should be interpreted as heritability on the mean across the evaluated years and replications, and not as a fixed property of the trait under any environmental condition. Its values therefore reflect the proportion of phenotypic variation attributable to permanent genotypic differences within the observed experimental window, after explicit separation of the genotype × year interaction.

#### 2.5.3. Comparison Between Bayesian Inference and REML

To evaluate the effect of estimation method and model specification on heritability estimates, the same data were analyzed by REML using the lme4 package [26] under two models: one excluding the genotype × year interaction component and one including it as a random effect. Heritability was calculated from the variance components estimated by REML in each case using the same algebraic expression adopted in the Bayesian analysis. The REML point estimates were then compared against the posterior distributions obtained from the full Bayesian model, which includes the G×Y component and provides credible intervals that reflect the true estimation uncertainty with two years of evaluation. This comparison allows two distinct effects to be disentangled: the specification bias introduced by omitting the genotype × year interaction, which results in the absorption of that variance into the genotypic component and systematic inflation of $H^{2}$, and the effect of the estimation method on the quantification of parametric uncertainty, which REML does not provide directly in interval form.

#### 2.5.4. Temporal Stability

Genotypic stability across the two years was assessed by two complementary criteria. Wricke’s ecovalence ($W_{i}$) was calculated as a univariate instability measure, quantifying the individual contribution of each genotype to the sum of squares of the G×Y interaction [27]. Genotypes with lower $W_{i}$ exhibit more predictable performance across years.

The Bayesian probability of consistent superiority was estimated as the proportion of posterior distribution samples in which the BLUP of a given genotype exceeded a pre-established selection intensity threshold (10%, 20%, and 25% above the overall mean) simultaneously in both evaluated years. The threshold was defined on the posterior predictive scale of the standardized BLUPs. Because BLUPs shrink toward the population mean, particularly for genotypes with lower effective replication, the applied thresholds are conservative relative to raw phenotypic means; this shrinkage effect tends to reduce probability estimates for genotypes with high uncertainty, which is a desirable property in the context of reliable candidate selection.

#### 2.5.5. Genetic Divergence

Phenotypic divergence among genotypes was characterized based on Bayesian BLUPs for % grain and FWM/bag, selected on the basis of their complementary informational content: % grain captures compositional efficiency while FWM/bag captures gravimetric conversion efficiency, together spanning the two main dimensions of processing performance identified in the multi-trait analysis (Section 3.4). The negative genetic correlation between these traits ($r_{G}=-0.731$) ensures that they are not redundant, maximizing the divergence information captured by the distance matrix. The remaining traits (FWM/GW and FVol/bag) are functionally derived from FWM/bag and would introduce near-collinear dimensions into the Mahalanobis distance, inflating distances without adding independent genetic information. The dissimilarity matrix was computed using Mahalanobis distance, which weights distances by the covariance structure among traits and is robust to differences in scale between characters. The conditioning of the covariance matrix was verified by the condition number κ, with a value of 22.5 indicating a well-conditioned system suitable for inversion.

Hierarchical clustering was performed using the UPGMA method (Unweighted Pair Group Method with Arithmetic Mean), selected based on a cophenetic correlation coefficient (CCC =0.813) superior to that of the Ward.D2 method (CCC =0.573). The dendrogram cutpoint was defined by the Mojena (1977) criterion with k=1.25 [28]. Principal component analysis (PCA) was conducted on the same standardized variables to visualize the divergence structure in two-dimensional space.

#### 2.5.6. Multi-Trait Model

The genetic correlation between % grain and FWM/bag was estimated using a multi-trait model with unstructured covariance (US) for the random effects of genotype, genotype × year, and residual, fitted by the AI-REML algorithm in the sommer package [10]. Three models were compared: diagonal (no covariances between traits), US for genotype with diagonal for genotype × year, and full US for all components. Model selection was performed by likelihood ratio test (LRT) with theoretical degrees of freedom. The full US structure was significantly superior to the diagonal model ($\chi^{2}=204.9$; df =3; $p=3.6\times 10^{-44}$) and to the model with covariance for genotype only ($\chi^{2}=36.3$; df =1; $p=1.7\times 10^{-9}$), and was adopted as the final model. With only two traits evaluated, factor analytic (FA) structures would involve more parameters than the US model (FA(1) =4 parameters; US =3) and were therefore not considered.

The multi-trait model was fitted using AI-REML (sommer) rather than the Bayesian MCMC framework (brms) employed for the univariate genetic parameter estimation. This choice was motivated by practical considerations: multi-trait Bayesian models with unstructured covariance matrices for three independent variance–covariance blocks (genotype, G×Y, and residual) require specifying LKJ or Wishart priors for correlation matrices, and convergence of such models with two years of data and 48 genotypes is computationally demanding and sensitive to prior specification [23]. The AI-REML estimator in sommer provided stable convergence and likelihood ratio-based model comparison, which was the primary objective of this analysis. We acknowledge that a fully Bayesian multi-trait framework would provide uncertainty quantification for the genetic correlations, which remains a limitation of the present analysis and a direction for future work as longer evaluation series become available.

All analyses were implemented in the R environment version 4.5.3 [29], using the packages brms [23], lme4 [26], loo [22], sommer [10], and ggplot2 for graphical visualization.

## 3. Results

### 3.1. Genetic Control and Variance Decomposition

**Figure 2:**
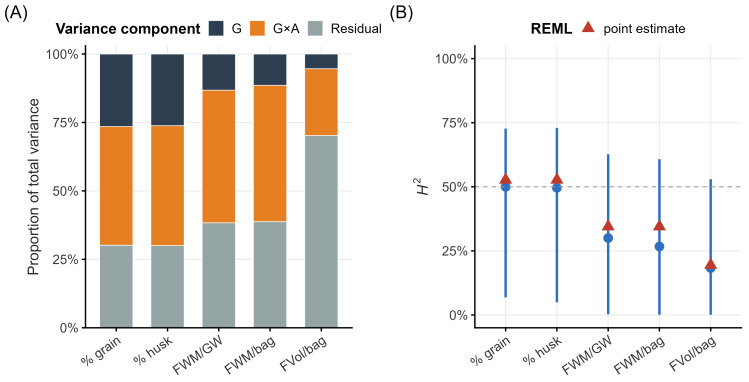
Variance decomposition and heritability estimates for five processing efficiency traits in *Coffea canephora*. (**A**) Proportion of total phenotypic variance attributed to genotype (G), genotype × year interaction (G×Y), and residual components, based on Bayesian posterior medians. (**B**) Broad-sense heritability ($H^{2}$) estimated by Bayesian inference (circle, with 95% credible interval) and by REML (triangle). The dashed line indicates $H^{2}=0.50$. The × symbol indicates a boundary solution in the REML estimate ($\sigma_{G}^{2}$ constrained to zero). Abbreviations: % grain = grain proportion; % husk = husk proportion; FWM/GW = fruit fresh mass per grain mass; FWM/bag = fruit fresh mass per bag; FVol/bag = fruit volume per bag.

The five traits retained for genetic analysis, grain proportion (% grain), husk proportion (% husk), fruit fresh mass per grain mass (FWM/GW), fruit fresh mass per bag (FWM/bag), and fruit volume per bag (FVol/bag) differed substantially in the magnitude and structure of genetic control (Table 2; Figure 2). The fruit volume-to-fresh mass ratio (FVol/FWM) showed no detectable genotypic variance ($H^{2}=0.02$ [95% CI: 0.00–0.17]) and was excluded from subsequent analyses.

**Figure 3:**
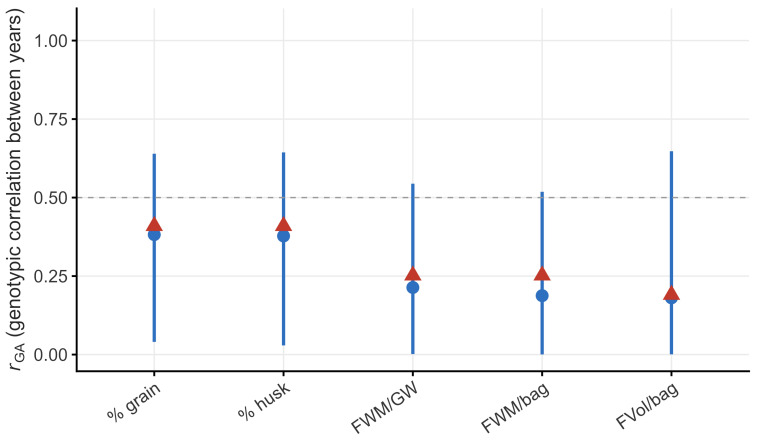
Genotypic correlation between years ($r_{GY}$) for five processing efficiency traits in *Coffea canephora*, estimated by Bayesian inference (circle, with 95% credible interval) and by REML (triangle). The dashed line indicates $r_{GY}=0.50$. Values below this threshold indicate that less than half of the genotypic variance is expressed consistently across years. Abbreviations: % grain = grain proportion; % husk = husk proportion; FWM/GW = fruit fresh mass per grain mass; FWM/bag = fruit fresh mass per bag; FVol/bag = fruit volume per bag.

For % grain and % husk, the genotypic component ($\sigma_{G}^{2}$) accounted for approximately 25–30% of total phenotypic variance, with posterior median heritabilities of 0.50 [0.07–0.73] and 0.50 [0.05–0.73], respectively. The genotype × year interaction ($\sigma_{GY}^{2}$) represented 43–46% of total variance for these two traits, exceeding the genotypic component in 78.8% of posterior samples. For FWM/bag and FWM/GW, the dominance of $\sigma_{GY}^{2}$ over $\sigma_{G}^{2}$ was even more pronounced, with $\sigma_{GY}^{2}$ exceeding $\sigma_{G}^{2}$ in 96.6% and 95.4% of posterior samples, respectively. Median heritabilities were correspondingly lower: 0.27 [0.001–0.61] for FWM/bag and 0.30 [0.003–0.63] for FWM/GW (Figure 2A; Table 2). The genotypic correlation between years ($r_{GY}$) ranged from 0.19 (FWM/bag) to 0.38 (% grain), indicating that a substantial portion of the genotypic signal is year-specific and does not reflect permanent genetic differences among genotypes (Figure 3).

REML-based $H^{2}$ estimates consistently exceeded the Bayesian posterior medians for the gravimetric traits. The most pronounced discrepancy was observed for FWM/bag (REML: 0.35 vs. Bayes: 0.27) and FVol/bag (REML: 0.19 vs. Bayes: 0.18). For FVol/FWM, the REML estimate collapsed to a boundary solution ($\sigma_{G}^{2}=0$), a condition that precludes uncertainty quantification and obscures the imprecision inherent in estimating variance components near zero (Figure 2B; Figure S1). The 95% credible intervals for $H^{2}$ spanned 0.60–0.72 units across all retained traits, reflecting the genuine imprecision of estimates derived from two years of evaluation, imprecision that point estimates systematically suppress.

### 3.2. Temporal Stability and Genotype × Year Interaction

**Figure 4:**
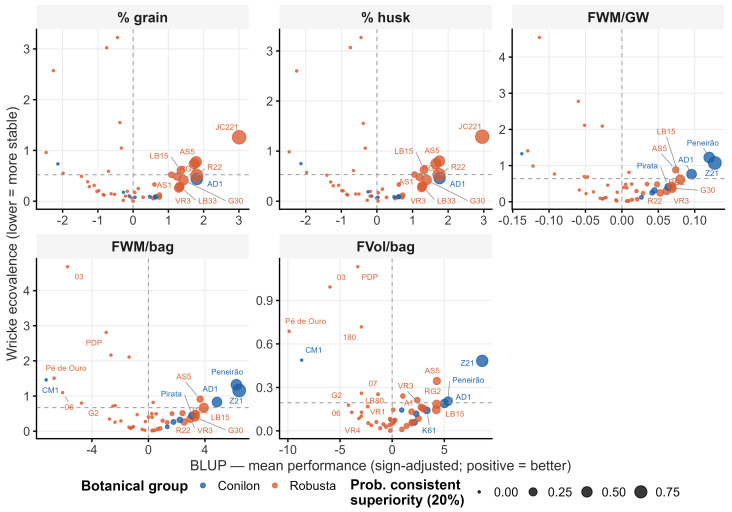
Mean performance and temporal stability of 48 *Coffea canephora* genotypes for five processing efficiency traits. The horizontal axis represents the mean BLUP adjusted by the trait directionality (positive values indicate higher efficiency). The vertical axis represents Wricke’s ecovalence (lower values indicate greater temporal stability). Point size is proportional to the Bayesian probability of consistent superiority at a 20% selection intensity. The horizontal dashed line indicates the panel mean ecovalence for each trait. Genotypes with a probability of consistent superiority exceeding 10%, or with performance at or above the 85th percentile of the panel, are identified by name.

**Figure 5:**
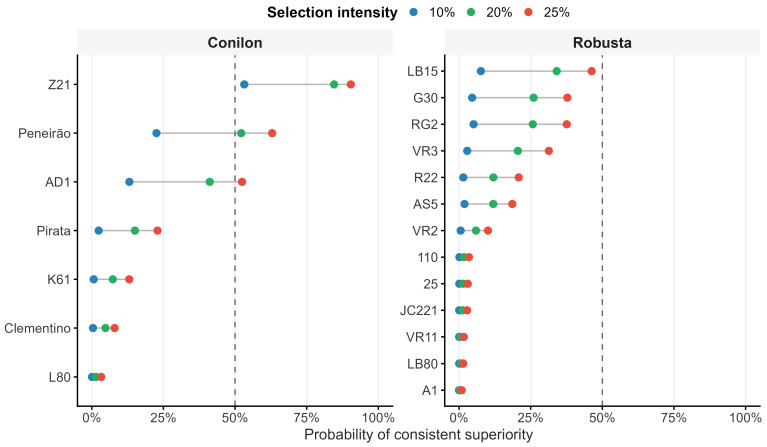
Bayesian probability of consistent superiority for fruit fresh mass per bag (FWM/bag) in the 20 genotypes with the highest maximum probability, stratified by botanical group. Each point represents the probability calculated under three selection intensities (10%, 20%, and 25% above the panel overall mean). The vertical dashed line indicates the 50% threshold. Genotypes to the right of the line show a high probability of maintaining superior performance in both evaluated years.

Temporal instability was pervasive across the evaluated panel. At a selection intensity of 20% above the overall mean, no genotype exhibited a Bayesian probability of consistent superiority exceeding 0.85 for more than one trait simultaneously, and only Z21 surpassed 0.50 for FWM/bag (prob. =0.846) (Figure 4). For the remaining genotypes, probabilities of consistent superiority for FWM/bag were below 0.45 regardless of botanical group, with LB15, G30, and RG2 showing the highest values among Robusta hybrids, ranging from 0.30 to 0.45 depending on the selection intensity applied (Figure 5). The proportion of genotypes that reversed their relative ranking between 2023 and 2024 ranged from 44% (% grain) to 52% (FWM/bag), and the inversion pattern was not restricted to materials of intermediate performance (Spearman’s ρ=−0.068, p=0.644 for the correlation between mean BLUP and rank-change magnitude for FWM/bag, confirming the absence of an association between performance level and ranking instability) (Figure S2).

Wricke’s ecovalence revealed an asymmetric pattern across traits. For % grain and % husk, genotypes with higher mean performance tended to concentrate greater contributions to the interaction, with JC221 accumulating markedly higher ecovalence than the remaining genotypes for both traits. For FWM/bag and FVol/bag, genotypes with lower gravimetric efficiency exhibited high instability regardless of mean performance, while genotypes with higher efficiency were distributed across a narrower ecovalence range. Z21 and Peneirão were positioned in the high-performance, low-ecovalence quadrant for FWM/bag and FVol/bag (Figure 5).

The two evaluation years differed substantially in environmental conditions (Figure S1): cumulative annual precipitation was 747 mm in 2023 and 1206 mm in 2024, with the harvest period (April–July) recording 280 mm and 171 mm respectively, and mean temperatures of 23.9 °C and 25.0 °C. These contrasts characterize the two years as genuinely distinct environments, providing biological context for the magnitude of the G×Y interaction detected, although a formal statistical association between climatic variables and interaction magnitude was not estimated in this single-location study.

### 3.3. Genotypes with Consistently Superior Performance

Despite the pervasive instability described in the preceding section, a subset of genotypes maintained a superior position in both evaluated years for FWM/bag. Complete results for performance, stability, and probability of consistent superiority across all five retained traits are provided in Table S1. Among Robusta hybrids, LB15 showed the highest probability of consistent superiority within the group (prob. =0.45 at 20% intensity), followed by G30 (0.43), RG2 (0.42), and VR3 (0.38). R22 and AS5 completed the set of Robusta hybrids with probabilities between 0.25 and 0.35 (Figure 5). Among Conilon genotypes, Z21 was the only one to exceed the 50% threshold (prob. =0.846 at 20% intensity), with Peneirão presenting a probability of 0.51 at 25% intensity. AD1 reached a probability of 0.50 at 25% intensity, ranking as the third Conilon genotype with consistently superior performance for FWM/bag (Figure 5).

The positioning of these genotypes in the performance-stability space was coherent between FWM/bag and FWM/GW, with VR3, G30, R22, and LB15 occupying the high-performance, below-average ecovalence quadrant for both traits (Figure 3). For % grain, the pattern differed: JC221 stood out as the genotype with the highest mean BLUP (deviation of +4.12 relative to the panel mean), but with markedly higher ecovalence than the remaining genotypes, indicating high but inconsistent performance across years (Figure 3). AD1 and AS5 were the only genotypes simultaneously allocated to the high-performance, low-ecovalence quadrant for both % grain and FWM/bag, characterizing a stable combined efficiency profile (Figure 3). BLUP values, relative ecovalence, and probability of consistent superiority for FWM/bag across all 48 genotypes are presented in Table 3.

**Figure 6:**
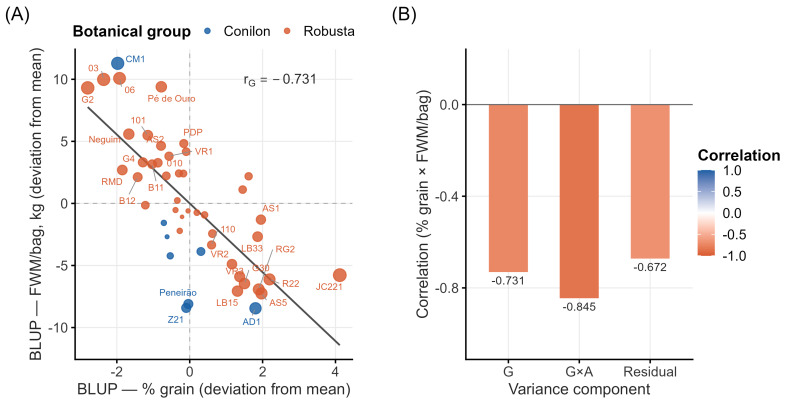
Multi-trait analysis for processing efficiency in 48 *Coffea canephora* genotypes. (**A**) Joint BLUPs for grain proportion (% grain) and fruit fresh mass per bag (FWM/bag), estimated by the unstructured covariance model (US). The line represents the linear regression between the two traits. Genotypes with an absolute multi-trait index exceeding 0.5 standard deviations are identified by name. (**B**) Correlations between % grain and FWM/bag by variance component: genotype (G), genotype × year interaction (G×Y), and residual. Values correspond to AI-REML estimates (sommer 4.4.4).

The multi-trait index, calculated from the joint BLUPs of % grain and FWM/bag under the unstructured covariance model, ranked JC221 first (index =2.02), followed by AD1 (1.44), AS5 (1.39), and R22 (1.36) (Figure 6A). The dissociation between JC221’s position in the multi-trait ranking and its high ecovalence for % grain reflects the partially independent nature of the two index components, an aspect quantified by the genetic correlation between traits presented in Section 3.4.

### 3.4. Genetic Divergence and Inter-Trait Correlations

**Figure 7:**
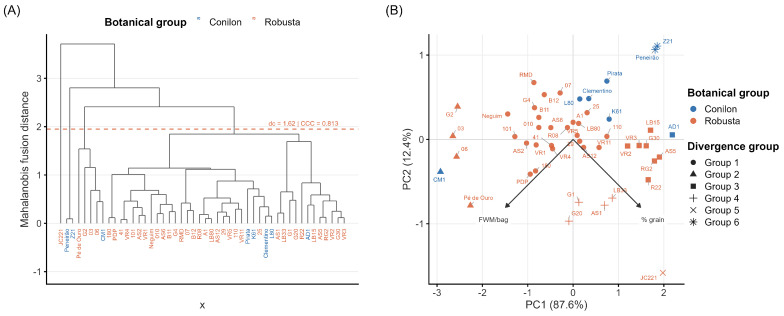
Genetic divergence among 48 *Coffea canephora* genotypes for processing efficiency. (**A**) Dendrogram obtained by the UPGMA method with Mahalanobis distance calculated from Bayesian BLUPs of grain proportion (% grain) and fruit fresh mass per bag (FWM/bag). The dashed line indicates the cutpoint defined by the Mojena criterion (k=1.25; $d_{c}=1.62$). Genotype labels are colored by botanical group (blue: Conilon; orange: Robusta). (**B**) Biplot of principal component analysis on the same standardized BLUPs. Vectors indicate the direction and magnitude of each trait’s contribution to the principal components. Symbols indicate the divergence group defined by the dendrogram.

Hierarchical clustering by the UPGMA method, using Mahalanobis distance calculated from Bayesian BLUPs of % grain and FWM/bag, yielded six distinct groups by the Mojena criterion (k=1.25; $d_{c}=1.62$), with a cophenetic correlation coefficient of 0.813, indicating satisfactory fit of the dendrogram to the original distance matrix (Figure 7A; Figure S4). The first principal component explained 87.6% of total variation, reflecting the predominantly unidimensional structure of divergence in processing efficiency across the evaluated panel (Figure 7B).

Within the limited set of processing efficiency traits evaluated here, botanical classification did not delimit the divergence groups. Of the six groups formed, only Group 6 was composed exclusively of Conilon genotypes, uniting Z21 and Peneirão with a profile of high gravimetric efficiency and above-average grain proportion. The remaining groups showed mixed composition or were formed exclusively by Robusta hybrids. Group 2, comprising CM1, G2, 03, 06, and Pé de Ouro, concentrated the genotypes with the lowest gravimetric efficiency in the panel, with CM1 being the only Conilon in this cluster. Group 3, formed by AD1 and LB15, brought together genotypes with high combined efficiency in both % grain and FWM/bag, with AD1 being the only Conilon in this group (Figure 7A,B).

Principal component analysis confirmed the partial dissociation between the two traits. JC221 occupied an isolated position in the biplot, with the highest positive PC1 score among all evaluated genotypes, simultaneously reflecting high grain proportion and moderate gravimetric efficiency, a pattern inconsistent with the general panel trend (Figure 4B). Z21 and Peneirão were positioned in the quadrant opposite to CM1 and G2 along PC1, while dispersion along PC2 was driven primarily by grain proportion independently of gravimetric efficiency.

The genetic correlation between % grain and FWM/bag estimated by the multi-trait model was $r_{G}=-0.731$ (point estimate from AI-REML; uncertainty quantification for this parameter is not available from the current model specification and is discussed in Section 4), indicating that genotypes with higher grain proportion tend genetically to require less fruit mass per bag. The correlation in the genotype × year interaction component was even stronger ($r_{G\times Y}=-0.845$), indicating that the temporal instability of both traits is co-regulated. The residual correlation was $r_{e}=-0.672$. The unstructured covariance model was significantly superior to the diagonal model, which assumes independence between traits (LRT: $\chi^{2}=204.9$; df =3; $p=3.6\times 10^{-44}$), and to the model with covariance restricted to the genotypic component (LRT: $\chi^{2}=36.3$; df =1; $p=1.7\times 10^{-9}$) (Figure 7B).

## 4. Discussion

### 4.1. Genetic Control and Variance Structure

The predominance of the genotype × year interaction over the genotypic component, with posterior probabilities $P(\sigma_{G\times Y}^{2}>\sigma_{G}^{2})$ ranging from 0.79 to 0.97 depending on the trait, is not unexpected when considering the physiological nature of the evaluated variables. Physical yield traits in *Coffea canephora*, particularly gravimetric ones, integrate assimilate allocation processes that occur within a relatively narrow developmental window and are sensitive to variation in water availability and temperature during fruit filling [8,12,30,31]. The 109 mm difference in cumulative precipitation between April and July of 2023 and 2024 coincides with this critical period, making an expressive genotype-by-year interaction biologically expected. However, since the experiment was conducted under supplemental irrigation, this association should not be interpreted as an isolated effect of precipitation. It is more appropriate to consider that the evaluated years differed with respect to a set of environmental conditions, including rainfall regime, mean temperature during the harvest period, and likely atmospheric demand, all of which may affect fruit maturation, filling, and physical composition.

The median heritability estimates reported here tend to be lower than point estimates from previous studies on *C. canephora* [2,5], although the 95% credible intervals are wide enough to overlap substantially with those previous estimates. This uncertainty is expected given the two-year evaluation window and should not be interpreted as evidence of fundamentally different genetic control; rather, it reflects the honest quantification of estimation imprecision that point estimates from single-year models cannot provide. In single-year analyses or in models that do not explicitly partition the G×Y interaction, the environmental sensitivity of genotypes remains confounded with the genotypic component, inflating heritability estimates relative to models that estimate $\sigma_{G}^{2}$ and $\sigma_{G\times Y}^{2}$ separately. This confounding is a well-documented consequence of incomplete variance partitioning in cultivar trials [6,7,32,33], and explains why single-year estimates for these traits in *C. canephora* frequently exceed those reported here. The boundary solution obtained by REML for FVol/FWM, in which $\sigma_{G}^{2}$ collapsed to zero, further illustrates how point estimates lacking explicit uncertainty quantification can precisely obscure the scenarios where that uncertainty is greatest.

The low genotypic correlation between years ($r_{GY}$ ranging from 0.19 for FWM/bag to 0.38 for % grain) indicates substantial reranking of genetic merit across years, with direct implications for selection. This level is lower than that observed in multi-environment studies with *C. canephora* [4,5], suggesting that physical yield traits, which depend strongly on developmental processes occurring within narrow physiological windows, may be more sensitive to interannual variation than cumulative traits such as yield per harvest. The implication is not that these traits are unsuitable for selection, but that their reliability depends on validation across a broader temporal and environmental range before definitive recommendations can be made.

### 4.2. Temporal Limitation

Evaluation over two years constitutes the main structural limitation of this study and should be interpreted considering what this temporal window represents biologically for *C. canephora* in northern Espírito Santo. The two harvest cycles (2023 and 2024) occurred under markedly distinct rainfall regimes, with total annual precipitation of 747 mm and 1206 mm, respectively, with a 109 mm difference during the critical fruit filling and maturation period (April–July). This contrast is not trivial: in Conilon coffee, the hydroclimatic regime during endosperm development can influence the formation of storage tissues and, consequently, the yield traits evaluated here [12,30,34]. In other words, the two years do not represent equivalent replications of the same environment, but genuine environmental contrasts that confer diagnostic power to the G×Y interaction detected. It should be noted, however, that while environmental contrast is sufficient for detecting the presence of G×Y interaction, it does not guarantee accurate estimation of the variance ratio $\sigma_{G\times Y}^{2}/\sigma_{G}^{2}$. With only two years, this ratio is likely overestimated relative to what would be obtained in a long-term multi-environment design, where additional years would partition residual and interaction variance more precisely [8,33].

Comparison with heritability estimates from the frequentist *C. canephora* literature must account for the fact that single-year studies, even when conducted with a larger number of replications, absorb G×Y variance into the genotypic component, potentially yielding $H^{2}$ estimates higher than those obtained when $\sigma_{G}^{2}$ and $\sigma_{G\times Y}^{2}$ are estimated separately [6,7,32,33]. The values reported here (0.27–0.50) do not necessarily reflect weaker genetic control of the evaluated traits, but rather the honest decomposition of variance that single-year models cannot disentangle. Figure 2B makes this distinction observable: REML estimates without G×Y for FWM/bag and % grain coincide with the upper bounds of the Bayesian credible intervals, not with their medians.

This does not imply that the estimated interaction is stable in a broad temporal sense, nor that two years are sufficient to characterize the long-term stabilization of variance components. However, this limitation should be interpreted with caution, as classical evidence advocating for larger numbers of evaluation years frequently derives from ANOVA-based approaches or models with restrictive assumptions of variance homogeneity across environments, assumptions that are relaxed in mixed and Bayesian models with explicit variance-covariance structures, the approach adopted here [8,35].

In this study, the primary limitation is not simply the reduced number of years, but the fact that two cycles may represent only a fraction of the culture’s temporal dynamics, particularly given the productive biennialism and autocorrelation between successive harvests, phenomena recognized in coffee that may affect the separation of year effects, G×Y interaction, and residual variation [8]. The present study therefore does not claim stabilization of variance components over the long term but provides an explicit probabilistic estimate of instability and its uncertainty within the observed temporal window, rather than implicitly assuming its absence by omitting the G×Y interaction [5,9]. Accordingly, the primary contribution of this study is not to define a definitive genetic hierarchy for processing efficiency, but to demonstrate that even under two contrasting years, the genotype × year interaction is sufficiently expressive to alter rankings and reduce the predictability of selection, evidence that justifies expanding evaluations to longer time series and additional environments.

### 4.3. Stability and Genotype Recommendation

The Bayesian probability of consistent superiority, defined as the proportion of MCMC samples in which a genotype exceeds the selection threshold simultaneously in both evaluated years, discriminates genotypes substantially differently from mean-based rankings. For FWM/bag at 20% selection intensity, Z21 showed a probability of 0.846, a value no Robusta genotype in the panel matched: the best performance in that group, LB15, reached 0.43 at the same intensity. This asymmetry does not reflect a difference in mean performance alone: Z21 combines a favorable BLUP with low Wricke ecovalence, while LB15 presents higher relative ecovalence and a probability that declines more sharply as selection intensity increases from 20% to 25% (Figure 5). The direct interpretation is that Z21 maintains its relative position across years more predictably than any Robusta genotype evaluated for this trait.

The behavior of VR3 warrants specific attention, as it is the only genotype consistently positioned in the high-performance, low-ecovalence quadrant across four of the five evaluated traits (% grain, % husk, FWM/bag, and FWM/GW, Figure 4). This multi-trait recommendation profile has a direct practical implication: in breeding programs without formal weighted selection indices, genotypes showing simultaneously favorable behavior across multiple processing efficiency traits reduce the risk of gains in one trait being accompanied by losses in another. The negative genetic correlation estimated between % grain and FWM/bag ($r_{G}=-0.731$) indicates that the opposition between these traits has a genetic basis in the evaluated panel, and VR3 represents one of the few genotypes in which this tension is attenuated.

The probability heatmap by year (Figure S2) reveals a pattern that warrants discussion beyond the ranking: the majority of genotypes with moderate probability in 2023 show near-zero probability in 2024, indicating that the more contrasting environment amplified genotypic separation, resulting in fewer genotypes with consistently superior performance. This behavior is consistent with what is observed in perennial crops under moderate water deficit, where genotypic variation in water-use efficiency during fruit filling tends to be expressed more intensely in drier years [36,37,38]. In the specific context of this experiment, the year with lower precipitation (2023) showed greater genotypic discriminating capacity in this dataset.

The absence of Conilon genotypes, beyond Z21, among the priority candidates for FWM/bag does not reflect systematic inferiority of that botanical group; it reflects the fact that the panel included proportionally more Robusta accessions (n=38 vs. n=10 Conilon). Peneirão and AD1, the two other Conilon genotypes with non-negligible probability (0.52 and 0.49 at 25% intensity, respectively), do not appear consistently across the remaining traits, limiting their recommendation for multi-trait selection. This panel imbalance should be considered when interpreting the predominance of Robusta in the favorable quadrants of Figure 4 and Figure 5. Accordingly, the superiority of Z21 should be understood as a probabilistic superiority conditioned on the evaluated genetic panel, the experimental site, and the two years assessed, an interpretation that is more conservative and more appropriate to the study’s experimental design, as it avoids extrapolating the observed stability to environmental contexts not yet tested.

It should be noted that the selection threshold was applied to posterior BLUP distributions rather than raw phenotypic means. The shrinkage inherent to BLUPs redistributes extreme observations toward the panel mean, which may reduce the estimated probability of consistent superiority for genotypes with fewer effective observations or higher residual variance. In the present design, all genotypes were evaluated with the same number of replications ($n_{r}=3$) and years ($n_{y}=2$), so differential shrinkage across genotypes is primarily driven by differences in the ratio $\sigma_{G}^{2}/\left(\sigma_{G}^{2}+\sigma_{GY}^{2}+\sigma_{e}^{2}\right)$. Genotypes with higher estimated genetic signal, such as Z21, are less affected by shrinkage, which partially explains their higher probability of consistent superiority relative to genotypes with similar raw means but higher ecovalence.

### 4.4. Genetic Correlation and Implications for Indirect Selection

The negative genetic correlation between % grain and FWM/bag ($r_{G}=-0.731$, US model, sommer) indicates that genotypes with a higher grain proportion in ripe fruit tend, on average, to require less cherry mass per processed bag, a biologically coherent pattern, since both traits capture related dimensions of the same underlying property: the efficiency of fruit-to-processed-bean conversion. The fact that this correlation is negative and of substantial magnitude is not trivial from a breeding perspective: it indicates that direct selection for % grain, operationally simpler as it does not require weighing processed lots, captures a substantial portion of the genetic variation in FWM/bag, opening the way for indirect selection strategies in panels where simultaneous measurement of all traits is logistically unfeasible [39,40].

The genetic correlation estimates reported here are point estimates from AI-REML and do not carry explicit uncertainty quantification. Although the likelihood ratio tests support the unstructured covariance structure over simpler alternatives, the precision of the individual correlation coefficients cannot be assessed without bootstrap confidence intervals or a fully Bayesian multi-trait model. Readers should therefore interpret the operational recommendations for indirect selection with appropriate caution, pending confirmation in designs with greater temporal replication.

From an operational standpoint, this result is relevant because grain proportion can be obtained from smaller samples with lower logistical demands than a direct estimate of the fruit mass required per processed bag. Thus, in early selection stages, % grain may function as an auxiliary trait for genotype screening, provided its association with FWM/bag is confirmed in additional evaluations.

The correlation in the G×Y interaction component was even more pronounced ($r_{G\times Y}=-0.845$), which requires careful interpretation. This result indicates that favorable interaction deviations for % grain tend to be associated with favorable deviations, in the opposite direction given the negative sign, for FWM/bag, revealing that the antagonistic association between the traits is even stronger in the interaction component than in the main genetic component. This has a direct implication for selection: genotypes superior in % grain in a given year also tend to show an advantage in FWM/bag in that environment, but the magnitude of this association may vary under different interannual conditions. The residual correlation ($r_{e}=-0.672$) is consistent with this pattern at the within-environment level as well.

The multi-trait BLUP scatter plot (Figure 6A) makes this pattern directly observable: genotypes JC221, AS1, and LB33 occupy the quadrant of high % grain and low FWM/bag, while G2, CM1, and accession 06 are concentrated in the opposite quadrant. Notably, Z21, the genotype with the highest probability of consistent superiority for FWM/bag, appears in the region of near-average % grain and below-average FWM/bag, a position compatible with a genotype that does not lead in grain proportion but maintains stable gravimetric efficiency across years. This dissociation between position in the multi-trait scatter and probability of consistent superiority is expected: the scatter reflects means across both years, while the Bayesian probability captures the consistency of relative position between years, representing complementary, not interchangeable, dimensions.

No genotype in this panel was identified that simultaneously combines high % grain, low FWM/bag, and a high probability of consistent superiority for both traits, suggesting that joint gains through direct selection may be constrained by the observed genetic correlation structure and interannual instability. Programs targeting simultaneous gain in both compositional and gravimetric efficiency will likely require weighted selection indices or additional evaluation cycles across a broader range of environments to identify genotypes that escape this pattern [41,42,43].

### 4.5. Genetic Divergence and Panel Structure

Hierarchical clustering by UPGMA with Mahalanobis distance on Bayesian BLUPs of % grain and FWM/bag yielded six groups by the Mojena criterion (k=1.25; $d_{c}=1.62$), with a cophenetic correlation coefficient of 0.813, a value indicating good dendrogram fit to the distance matrix and exceeding the threshold commonly considered adequate for the interpretation of hierarchical clusters [44,45]. The first principal component explained 87.6% of total variation, indicating that the divergence structure in the panel is predominantly unidimensional for the two evaluated traits. Genotypes are distributed mainly along a gradient separating material with higher gravimetric efficiency and higher grain proportion (positive PC1) from those with lower efficiency in both traits (negative PC1).

Within the limited set of processing efficiency traits evaluated here, botanical classification did not delimit the divergence groups, a result that requires careful interpretation, as it might superficially suggest that Conilon and Robusta are indistinguishable for processing efficiency under this restricted trait space. This result does not indicate an absence of differentiation between Conilon and Robusta, but rather that botanical classification alone does not adequately explain the divergence observed for these specific processing efficiency traits. In other words, the variation relevant to these traits occurs both between and within botanical groups. What the dendrogram reveals is more specific: of the six groups formed, five are of mixed composition or composed exclusively of Robusta hybrids, and only Group 6 brought together Conilon genotypes exclusively (Z21 and Peneirão) positioned at the positive extreme of PC1 with the highest combined efficiency scores in the panel. CM1, the only Conilon outside Group 6, was assigned to Group 2 alongside the lowest-efficiency genotypes in the panel (G2, 03, 06, and Pé de Ouro), indicating that variation in processing efficiency within the Conilon group is broad enough to place genotypes in opposite divergence clusters. This pattern is consistent with what is known about the genetic structure of *C. canephora*: the Conilon/Robusta distinction reflects origin and population structure but does not imply phenotypic uniformity within each group for specific quantitative traits [46,47].

Group 3, formed exclusively by AD1 and LB15, warrants attention for the profile it represents: both genotypes combine high grain proportion and above-average gravimetric efficiency, with AD1 being the only Conilon in this cluster. Their position in the biplot (positive PC1 quadrant, PC2 near zero) suggests jointly favorable performance across both traits, without the deviation observed for JC221, which occupies an isolated position at the positive extreme of PC1 with a strong PC2 contribution, indicating a distinct profile within the set of superior genotypes, a pattern already discussed in the preceding section.

The Mahalanobis distance between the extreme groups, Group 6 (Z21, Peneirão) and Group 2 (CM1, G2, 03, 06, Pé de Ouro), represents the greatest divergence observed in the panel. Crosses between genotypes from more distant groups are frequently associated with a higher probability of generating transgressive segregants [48,49], although the negative genetic correlation between the two traits ($r_{G}=-0.731$) indicates that simultaneous gains in both directions will depend on favorable recombination at loci with pleiotropic effects or in linkage disequilibrium. The structure of six groups with substantial divergence amplitude suggests that the panel, despite being limited to 48 genotypes, contains sufficient variability to sustain recurrent selection cycles focused on processing efficiency.

The recommendations derived from this study are intentionally probabilistic. Z21, VR3, AD1, and LB15 were identified as priority candidates for processing efficiency in *C. canephora*, but their position in this panel does not warrant a definitive cultivar recommendation; it supports a hypothesis of consistent superiority, testable in multi-location, multi-year networks with a broader range of environments. This distinction is not rhetorical: it follows from the model adopted, which quantifies the probability of maintaining relative position across crop seasons, rather than assuming that the ranking from a local experiment reflects a permanent genetic hierarchy. The value of this type of information for breeding programs lies not in immediate selection, but in the rational prioritization of candidates for subsequent evaluation stages, reducing the risk of advancing unstable genotypes through the selection cycle.

## 5. Conclusions

In this single-location, two-year study, the genotype × year interaction was the predominant variance component for all processing efficiency traits evaluated in *Coffea canephora*, with posterior probabilities $P(\sigma_{G\times Y}^{2}>\sigma_{G}^{2})$ ranging from 0.79 to 0.97, demonstrating that temporal stability must be considered alongside mean performance in selection decisions. Bayesian heritability estimates ($H^{2}$) were lower than REML estimates, a result consistent with the explicit partitioning of interaction variance in the model. The low genotypic correlations between years ($r_{GY}$) indicated substantial reranking of genetic merit and limited the reliability of selection based exclusively on single-year evaluations. Spearman rank correlations between genotypic values in 2023 and 2024 confirmed this instability: ρ=0.08 for FWM/GW, ρ=0.87 for % grain, ρ=0.87 for % husk, ρ=0.97 for FWM/bag, and ρ=0.99 for FVol/bag, indicating that rank consistency varied substantially across traits and was particularly low for FWM/GW.

Probability of consistent superiority analysis identified Z21 and VR3 as promising candidates for processing efficiency in the evaluated panel, particularly for combining favorable performance with greater temporal consistency. The negative genetic correlation between % grain and FWM/bag indicates a favorable association between higher grain proportion and lower fruit mass required per processed bag, suggesting potential for indirect selection. The genetic divergence observed, not fully explained by the Conilon/Robusta botanical classification, indicates the availability of useful variability for recombination in recurrent selection programs. Nevertheless, the recommendation of these genotypes must be conditioned on validation in multi-location, multi-year networks.

## Figures and Tables

**Table 1:** *Coffea canephora* genotypes evaluated, with identification of botanical group.

Genotype	Group	Genotype	Group	Genotype	Group
AD1	Conilon	010	Robusta hybrid	G20	Robusta hybrid
Clementino	Conilon	03	Robusta hybrid	G30	Robusta hybrid
CM1	Conilon	06	Robusta hybrid	JC221	Robusta hybrid
K61	Conilon	07	Robusta hybrid	LB15	Robusta hybrid
L80	Conilon	25	Robusta hybrid	LB33	Robusta hybrid
Peneirão	Conilon	29	Robusta hybrid	LB80	Robusta hybrid
Pirata	Conilon	41	Robusta hybrid	Neguim	Robusta hybrid
Z21	Conilon	101	Robusta hybrid	PDP	Robusta hybrid
A1	Hybrid ^1^	110	Robusta hybrid	Pé de Ouro	Robusta hybrid
AS1	Robusta hybrid	180	Robusta hybrid	R08	Robusta hybrid
AS2	Robusta hybrid	AS5	Robusta hybrid	R22	Robusta hybrid
AS6	Robusta hybrid	AS12	Robusta hybrid	RG2	Robusta hybrid
B11	Robusta hybrid	G1	Robusta hybrid	RMD	Robusta hybrid
B12	Robusta hybrid	G2	Robusta hybrid	VR1	Robusta hybrid
VR2	Robusta hybrid	VR3	Robusta hybrid	VR4	Robusta hybrid
VR5	Robusta hybrid	VR11	Robusta hybrid	G4	Robusta hybrid

^1^ Genotype originally classified as Conilon from Espírito Santo, reclassified as an interspecific hybrid (group G2) by genomic analysis with SNP markers [18].

**Table 2:** Posterior median estimates and 95% credible intervals (95% CI) of variance components and genetic parameters for five processing efficiency traits in 48 *Coffea canephora* genotypes evaluated over two years (2023–2024) in Jaguaré, Espírito Santo, Brazil.

Trait	$\sigma_{G}^{2}$	$\sigma_{\mathrm{GY}}^{2}$	$\sigma_{e}^{2}$	$H_{\mathrm{mean}}^{2}$ (95% CI)	$r_{\mathrm{GY}}$ (95% CI)	${\mathrm{CV}}_{g}$ (%)
% grain	3.27	5.36	3.72	0.50 (0.07–0.73)	0.38 (0.04–0.64)	3.27
% husk	3.25	5.43	3.72	0.50 (0.05–0.73)	0.38 (0.03–0.64)	4.03
FWM/GW	0.019	0.068	0.054	0.30 (0.003–0.63)	0.21 (0.002–0.54)	3.66
FWM/bag	57.8	252	196	0.27 (0.001–0.61)	0.19 (0.001–0.52)	3.40
FVol/bag	120	545	1570	0.18 (0.001–0.53)	0.18 (0.001–0.65)	3.08

$\sigma_{G}^{2}$: genotypic variance; $\sigma_{GY}^{2}$: genotype × year interaction variance; $\sigma_{e}^{2}$: residual variance; $H_{\mathrm{mean}}^{2}$: broad-sense heritability estimated on a mean basis across years and replications (not individual-plant heritability); $r_{GY}$: genotypic correlation between years; $CV_{g}$: genotypic coefficient of variation. Parameters estimated by Bayesian inference (brms; 4 chains × 4000 iterations; Student-*t*(3, 0, 2.5) priors). The FVol/FWM trait was excluded due to absence of detectable genotypic variance ($H^{2}=0.02$ [95% CI: 0.00–0.17]).

**Table 3:** Mean performance, temporal stability, and probability of consistent superiority for fruit fresh mass per bag (FWM/bag) in 48 *Coffea canephora* genotypes evaluated over two years (2023–2024) in Jaguaré, Espírito Santo, Brazil. BLUP: best linear unbiased predictor (deviation from overall mean, kg bag^−1^). For FWM/bag, negative BLUP values indicate favorable performance, as lower fruit mass per processed bag represents greater processing efficiency. Performance rank 1 corresponds to the genotype requiring the least fruit mass per bag (most efficient). The same directionality applies to FWM/GW and FVol/bag, whereas for % grain, higher (positive) BLUPs are favorable. Wricke (%$W_{i}$): relative Wricke ecovalence (lower = greater temporal stability). Pr(consistent superiority): Bayesian probability of maintaining superior position in both evaluated years at a selection intensity of 20% above the overall mean. Divergence group: group formed by the UPGMA method with Mahalanobis distance on % grain and FWM/bag BLUPs. Ranks: 1 = best. Genotypes in bold have Pr(consistent superiority) ≥0.50.

Genotype	Bot. Group	Div. Group	BLUP	Wricke	Pr(sup.)	Perf. Rank	Stab. Rank	Comp. Rank
A1	Robusta	Group 1	−0.41	0.1	0.003	21	2	11.5
JC221	Robusta	Group 5	−0.83	0.2	0.012	18	6	12.0
LB80	Robusta	Group 1	−0.65	0.2	0.007	19	5	12.0
VR5	Robusta	Group 1	−0.27	0.1	0.002	23	1	12.0
R22	Robusta	Group 3	−2.53	0.8	0.120	10	16	13.0
L80	Conilon	Group 1	−1.33	0.4	0.015	15	11	13.0
Clementino	Conilon	Group 1	−1.77	0.8	0.048	13	15	14.0
25	Robusta	Group 1	−1.33	0.8	0.015	16	13	14.5
G30	Robusta	Group 3	−3.31	1.2	0.260	7	24	15.5
VR3	Robusta	Group 3	−2.98	1.1	0.205	9	22	15.5
B12	Robusta	Group 1	0.26	0.1	0.000	28	3	15.5
K61	Conilon	Group 1	−2.22	1.0	0.073	12	20	16.0
RMD	Robusta	Group 1	0.45	0.2	0.000	29	4	16.5
RG2	Robusta	Group 3	−3.32	1.5	0.258	6	28	17.0
Pirata	Conilon	Group 1	−3.10	1.4	0.151	8	26	17.0
VR11	Robusta	Group 1	−1.01	0.9	0.007	17	19	18.0
LB15	Robusta	Group 3	−3.93	2.1	0.341	4	33	18.5
41	Robusta	Group 1	1.23	0.3	0.000	33	7	20.0
AD1	Conilon	Group 3	−4.86	2.6	0.412	3	38	20.5
**Z21**	**Conilon**	**Group 6**	−6.42	**3.6**	**0.846**	**1**	**41**	**21.0**
VR4	Robusta	Group 1	1.26	0.3	0.000	34	8	21.0
B11	Robusta	Group 1	1.11	0.4	0.000	32	10	21.0
VR2	Robusta	Group 3	−2.42	1.6	0.059	11	32	21.5
G4	Robusta	Group 1	1.07	0.4	0.000	31	12	21.5
**Peneirão**	**Conilon**	**Group 6**	−6.23	**4.1**	**0.521**	**2**	**42**	**22.0**
AS5	Robusta	Group 3	−3.66	2.8	0.119	5	39	22.0
AS1	Robusta	Group 4	0.22	0.8	0.000	27	17	22.0
110	Robusta	Group 1	−1.67	1.6	0.017	14	31	22.5
G1	Robusta	Group 4	1.38	0.4	0.000	36	9	22.5
AS12	Robusta	Group 1	−0.26	1.2	0.000	24	23	23.5
07	Robusta	Group 1	−0.54	1.5	0.001	20	29	24.5
R08	Robusta	Group 1	0.00	1.3	0.001	26	25	25.5
AS2	Robusta	Group 1	2.37	0.8	0.000	39	14	26.5
29	Robusta	Group 1	−0.24	1.5	0.001	25	30	27.5
VR1	Robusta	Group 1	1.95	0.9	0.000	37	18	27.5
AS6	Robusta	Group 1	0.80	1.5	0.000	30	27	28.5
LB33	Robusta	Group 4	−0.33	2.5	0.000	22	37	29.5
101	Robusta	Group 1	2.76	1.1	0.000	42	21	31.5
G20	Robusta	Group 4	2.35	2.3	0.000	38	35	36.5
Neguim	Robusta	Group 1	2.52	2.2	0.000	40	34	37.0
010	Robusta	Group 1	1.37	6.5	0.000	35	45	40.0
G2	Robusta	Group 2	4.74	2.5	0.000	44	36	40.0
06	Robusta	Group 2	6.08	3.4	0.000	46	40	43.0
180	Robusta	Group 1	2.65	6.7	0.000	41	46	43.5
PDP	Robusta	Group 1	2.99	8.7	0.000	43	47	45.0
CM1	Conilon	Group 2	7.24	4.5	0.000	48	43	45.5
Pé de Ouro	Robusta	Group 2	6.68	4.7	0.000	47	44	45.5
03	Robusta	Group 2	5.73	14.5	0.000	45	48	46.5

## Data Availability

The R scripts used for all statistical analyses reported in this study are publicly available at https://github.com/juniorherenio/canephora-processing-efficiency (accessed on 1 June 2026). Raw data are not publicly available due to their origin in an active breeding program; requests for data access may be directed to the corresponding author.

## Supplementary Materials

The following supporting information can be downloaded at: https://www.mdpi.com/article/10.3390/biology15120899/s1, Figure S1: Comparison of variance components estimated by Bayesian inference (Bayes) and restricted maximum likelihood (REML) for six traits in *Coffea canephora*; Figure S2: Individual superiority probability by year for 48 *Coffea canephora* genotypes at a selection intensity of 20% above the overall mean; Figure S3: Test of the year × block interaction by the difference in Akaike Information Criterion (ΔAIC) for five *Coffea canephora* traits; Figure S4: Mahalanobis distance matrix among 48 *Coffea canephora* genotypes calculated from Bayesian BLUPs of % grain and FWM/bag; Table S1: Mean performance, temporal stability, and probability of consistent superiority for five processing efficiency traits in 48 *Coffea canephora* genotypes evaluated over two years (2023–2024).

## Abbreviations

The following abbreviations are used in this manuscript:

BLUP	Best linear unbiased predictor
MCMC	Markov chain Monte Carlo
REML	Restricted maximum likelihood
HMC-NUTS	Hamiltonian Monte Carlo with No-U-Turn Sampler
WAIC	Watanabe–Akaike Information Criterion
LOO	Leave-one-out cross-validation
ELPD	Expected log pointwise predictive density
AIC	Akaike Information Criterion
LRT	Likelihood ratio test
UPGMA	Unweighted Pair Group Method with Arithmetic Mean
PCA	Principal component analysis
CCC	Cophenetic correlation coefficient
G×Y	Genotype × year interaction
G×E	Genotype × environment interaction
$H^{2}$	Broad-sense heritability on a mean basis
$r_{GY}$	Genotypic correlation between years
$CV_{g}$	Genotypic coefficient of variation
$W_{i}$	Wricke ecovalence
% grain	Grain proportion
% husk	Husk proportion
FWM/GW	Fruit fresh mass per grain mass
FWM/bag	Fruit fresh mass per bag
FVol/bag	Fruit volume per bag
FVol/FWM	Fruit volume-to-fresh mass ratio
US	Unstructured covariance
FA	Factor analytic
AI-REML	Average information restricted maximum likelihood
SNP	Single nucleotide polymorphism
UFES	Universidade Federal do Espírito Santo
FAPES	Fundação de Amparo à Pesquisa e Inovação do Espírito Santo
CNPq	Conselho Nacional de Desenvolvimento Científico e Tecnológico
