# Supplementary material for: Genetic Variation in Fruit-to-Grain Conversion Efficiency in Coffea canephora: Heritability, Temporal Instability, and Divergence in Robusta Hybrids and Conilon
Source: Biology (Basel). 2026 Jun 8;15(12):899. doi: 10.3390/biology15120899 (PMC13295605; doi:10.3390/biology15120899)
Supplement: Supplementary file 1 [file biology-15-00899-s001.zip › biology-4337007-supplementary.pdf]

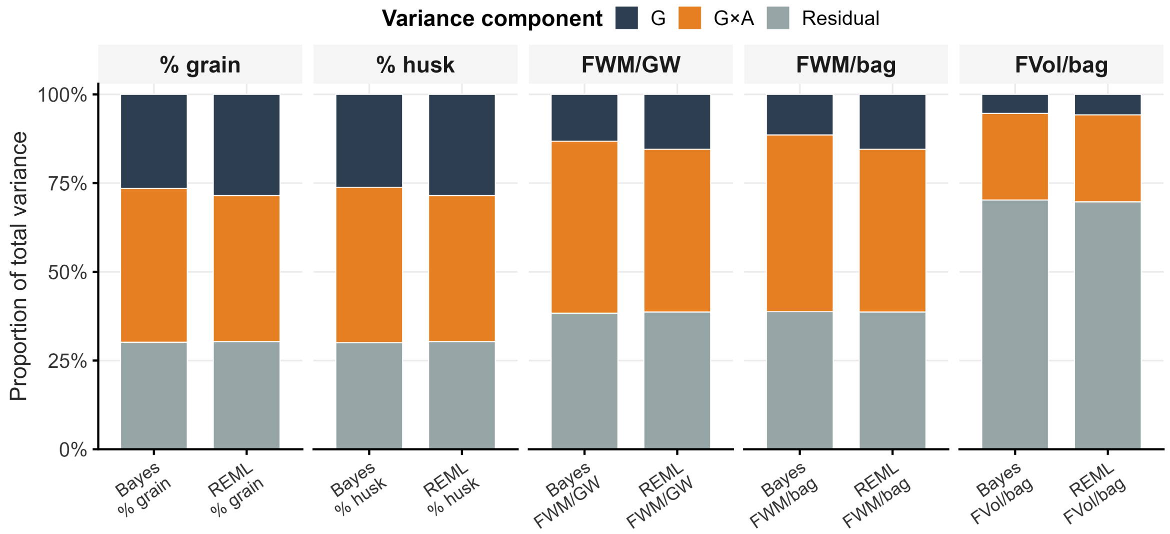

Figure S1: Comparison of variance components estimated by Bayesian inference (Bayes) and restricted maximum likelihood (REML) for six traits in *Coffea canephora*. Bars represent the proportion of total phenotypic variance attributed to the genotype (G), genotype × year interaction (G×Y), and residual components. Bayesian estimates correspond to posterior medians; REML estimates are point estimates without uncertainty quantification. The discrepancy between methods is most pronounced for FWM/bag and FWM/GW, traits for which the REML solution produces greater absorption of G×Y variance into the genotypic component. Abbreviations: % grain = grain proportion; % husk = husk proportion; FWM/GW = fruit fresh mass per grain mass; FWM/bag = fruit fresh mass per bag; FVol/bag = fruit volume per bag; FVol/FWM = fruit volume-to-fresh mass ratio.

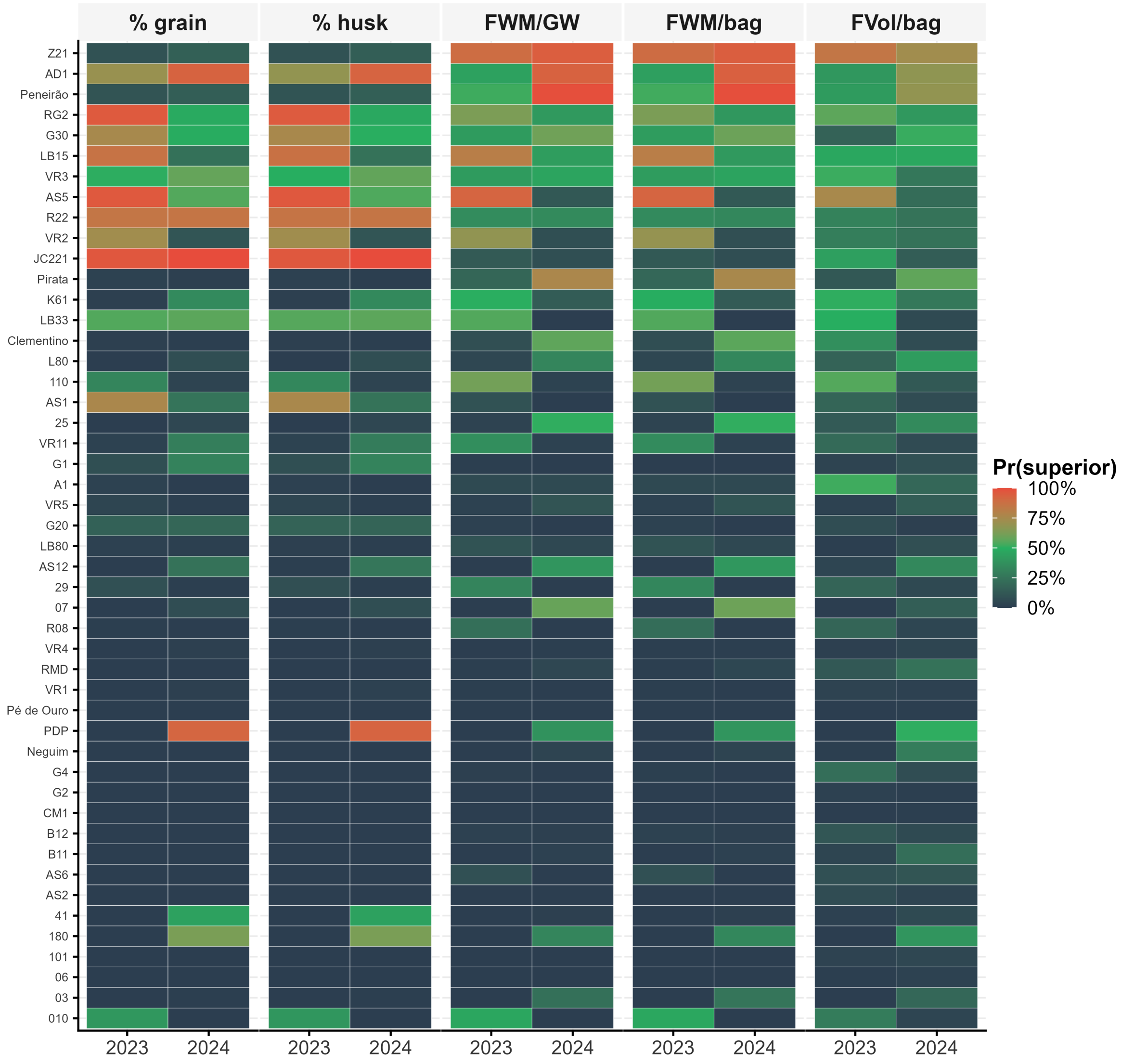

Figure S2: Individual superiority probability by year for 48 *Coffea canephora* genotypes at a selection intensity of 20% above the overall mean. Each cell represents the probability of a genotype exceeding the selection threshold in the indicated year, derived from posterior distribution samples (MCMC). Genotypes are ordered by their probability of consistent superiority (both years simultaneously). The color scale ranges from low probability (dark blue) to high probability (red). The dissociation between cells of the same genotype across 2023 and 2024 reflects the magnitude of the genotype × year interaction for each trait. Abbreviations: % grain = grain proportion; % husk = husk proportion; FWM/GW = fruit fresh mass per grain mass; FWM/bag = fruit fresh mass per bag; FVol/bag = fruit volume per bag.

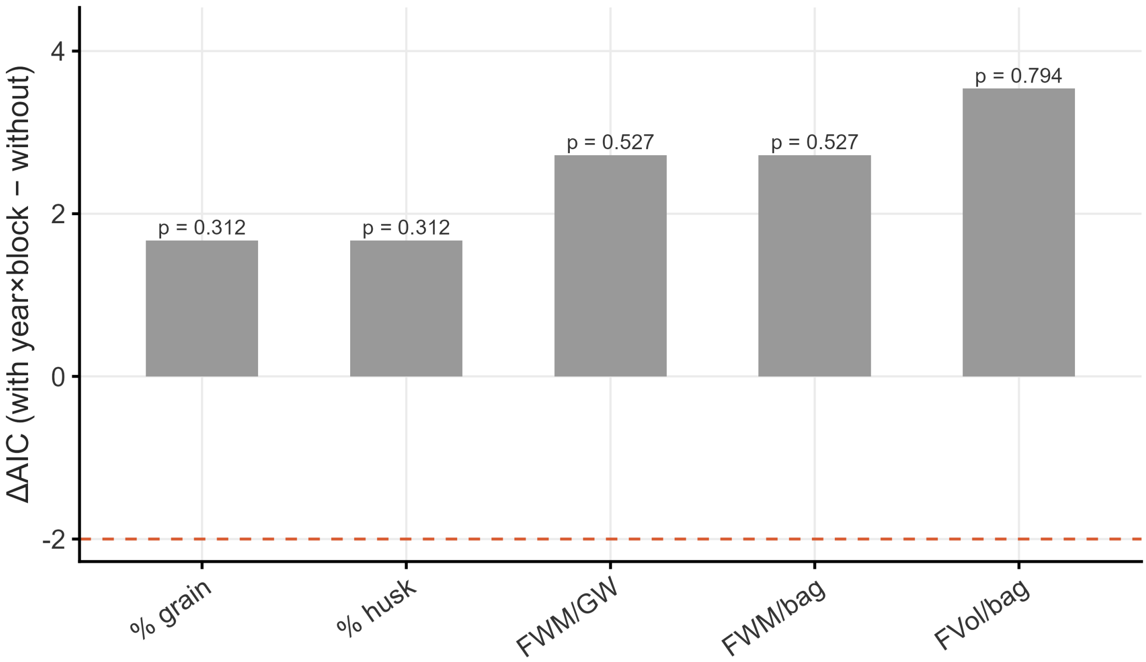

Figure S3: Test of the year × block interaction by the difference in Akaike Information Criterion (ΔAIC) between models with and without this term in the fixed structure, for five *Coffea canephora* traits. Positive ΔAIC values indicate that inclusion of the year × block term worsens model fit. The dashed line indicates $\Delta\text{AIC} = -2$, the conventional threshold for effect relevance. p-values correspond to likelihood ratio tests (LRT). The year × block term was not significant for any trait, justifying its exclusion from the final model. Abbreviations: % grain = grain proportion; % husk = husk proportion; FWM/GW = fruit fresh mass per grain mass; FWM/bag = fruit fresh mass per bag; FVol/bag = fruit volume per bag.

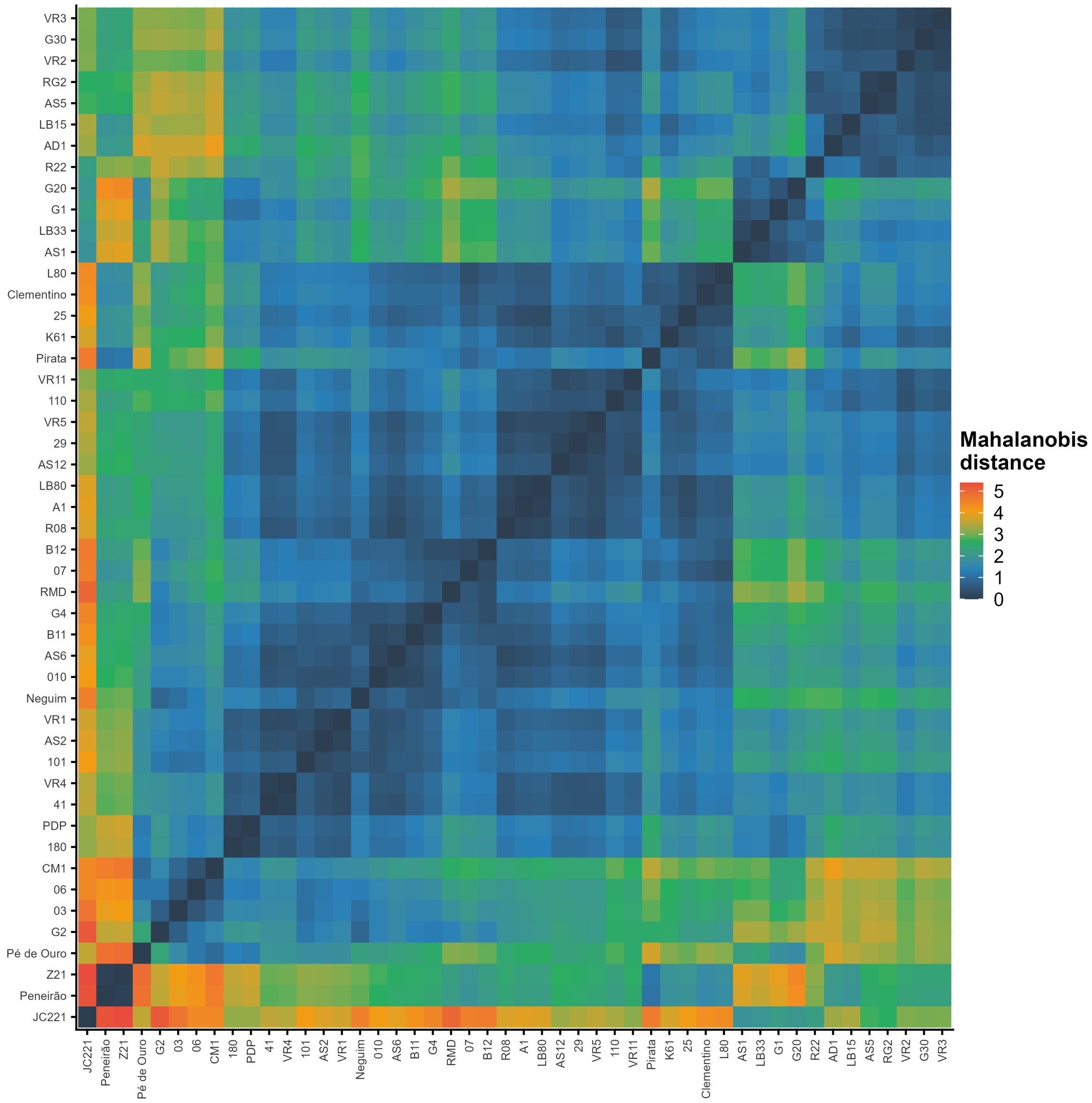

Figure S4: Mahalanobis distance matrix among 48 *Coffea canephora* genotypes, calculated from Bayesian BLUPs of grain proportion (% grain) and fruit fresh mass per bag (FWM/bag). Genotypes are ordered according to the UPGMA dendrogram sequence (Figure 7A of the main text), so that genotypes belonging to the same divergence group form low-distance blocks (dark blue cells) along the diagonal. High distances (red) indicate pairs of genotypes with the most contrasting processing efficiency profiles, representing parental combinations with the greatest potential for exploiting variability in targeted crosses.

Table S1: Mean performance, temporal stability, and probability of consistent superiority for five processing efficiency traits in 48 *Coffea canephora* genotypes evaluated over two years (2023–2024) in Jaguaré, Espírito Santo, Brazil. For definitions of columns, see Table 3 caption in the main text.

**Trait**	**Genotype**	**Bot.**	**Div. grp**	**BLUP**	**Wricke**	**Pr(sup.)**	**Perf.**	**Stab.**	**Comp.**
% grain	G1	Robusta	Group 4	0.742	0.3	0.029	13	7	10.0
% grain	VR11	Robusta	Group 1	0.570	0.2	0.008	17	3	10.0
% grain	Z21	Conilon	Group 6	0.638	0.3	0.018	14	8	11.0
% grain	25	Robusta	Group 1	0.000	0.0	0.000	23	1	12.0
% grain	Peneirão	Conilon	Group 6	0.635	0.4	0.018	15	10	12.5
% grain	AS12	Robusta	Group 1	0.282	0.3	0.001	19	6	12.5
% grain	G20	Robusta	Group 4	0.746	0.5	0.031	12	14	13.0
% grain	Pirata	Conilon	Group 1	0.052	0.3	0.000	21	5	13.0
% grain	K61	Conilon	Group 1	0.474	0.3	0.004	18	9	13.5
% grain	LB80	Robusta	Group 1	−0.049	0.3	0.000	24	4	14.0
% grain	VR4	Robusta	Group 1	−0.280	0.1	0.000	29	2	15.5
% grain	VR3	Robusta	Group 3	1.295	1.0	0.303	9	24	16.5
% grain	AD1	Conilon	Group 3	1.805	1.8	0.662	3	31	17.0
% grain	LB33	Robusta	Group 4	1.320	1.2	0.314	8	26	17.0
% grain	R22	Robusta	Group 3	1.805	2.0	0.736	2	34	18.0
% grain	G30	Robusta	Group 3	1.429	1.7	0.379	6	30	18.0
% grain	Clementino	Conilon	Group 1	−0.110	0.4	0.000	25	12	18.5
% grain	R08	Robusta	Group 1	−0.217	0.4	0.000	27	11	19.0
% grain	VR5	Robusta	Group 1	0.042	0.7	0.000	22	18	20.0
% grain	AS1	Robusta	Group 4	1.250	1.9	0.196	10	32	21.0
% grain	29	Robusta	Group 1	0.147	1.0	0.000	20	23	21.5
% grain	110	Robusta	Group 1	0.604	1.3	0.010	16	28	22.0
% grain	JC221	Robusta	Group 5	3.008	5.0	0.970	1	44	22.5
% grain	AS5	Robusta	Group 3	1.786	3.1	0.531	4	41	22.5
% grain	RG2	Robusta	Group 3	1.735	2.9	0.466	5	40	22.5
% grain	LB15	Robusta	Group 3	1.354	2.4	0.210	7	38	22.5
% grain	VR2	Robusta	Group 3	1.085	2.1	0.083	11	35	23.0
% grain	A1	Robusta	Group 1	−0.165	0.8	0.000	26	21	23.5
% grain	L80	Conilon	Group 1	−0.274	0.7	0.000	28	19	23.5
% grain	AS6	Robusta	Group 1	−0.525	0.5	0.000	33	16	24.5
% grain	AS2	Robusta	Group 1	−0.822	0.5	0.000	37	13	25.0
% grain	VR1	Robusta	Group 1	−0.625	0.6	0.000	34	17	25.5
% grain	B11	Robusta	Group 1	−0.850	0.5	0.000	38	15	26.5
% grain	G4	Robusta	Group 1	−1.027	0.8	0.000	40	20	30.0
% grain	B12	Robusta	Group 1	−0.976	0.8	0.000	39	22	30.5
% grain	Pé de Ouro	Robusta	Group 2	−1.241	1.1	0.000	42	25	33.5
% grain	101	Robusta	Group 1	−1.104	1.2	0.000	41	27	34.0
% grain	07	Robusta	Group 1	−0.699	2.4	0.000	35	37	36.0
% grain	RMD	Robusta	Group 1	−1.290	1.5	0.000	43	29	36.0
% grain	41	Robusta	Group 1	−0.334	4.2	0.000	30	43	36.5
% grain	180	Robusta	Group 1	−0.376	6.2	0.000	31	45	38.0
% grain	Neguim	Robusta	Group 1	−1.462	2.0	0.000	44	33	38.5
% grain	PDP	Robusta	Group 1	−0.443	12.9	0.000	32	48	40.0
% grain	06	Robusta	Group 2	−1.985	2.2	0.000	45	36	40.5
% grain	010	Robusta	Group 1	−0.750	12.1	0.000	36	47	41.5
% grain	CM1	Conilon	Group 2	−2.134	2.9	0.000	46	39	42.5
% grain	G2	Robusta	Group 2	−2.467	3.8	0.000	48	42	45.0
% grain	03	Robusta	Group 2	−2.262	10.3	0.000	47	46	46.5
% husk	VR11	Robusta	Group 1	−0.514	0.2	0.007	17	3	10.0

Continued on next page

Table S1 continued

Trait	Genotype	Bot.	Div. grp	BLUP	Wricke	Pr(sup.)	Perf.	Stab.	Comp.
% husk	G1	Robusta	Group 4	−0.708	0.3	0.029	13	9	11.0
% husk	Z21	Conilon	Group 6	−0.608	0.3	0.015	16	7	11.5
% husk	25	Robusta	Group 1	−0.031	0.0	0.000	22	1	11.5
% husk	G20	Robusta	Group 4	−0.724	0.5	0.038	12	13	12.5
% husk	Peneirão	Conilon	Group 6	−0.609	0.4	0.019	15	10	12.5
% husk	AS12	Robusta	Group 1	−0.270	0.3	0.001	19	6	12.5
% husk	K61	Conilon	Group 1	−0.487	0.3	0.003	18	8	13.0
% husk	Pirata	Conilon	Group 1	−0.028	0.3	0.000	23	5	14.0
% husk	LB80	Robusta	Group 1	0.014	0.3	0.000	24	4	14.0
% husk	VR4	Robusta	Group 1	0.272	0.1	0.000	29	2	15.5
% husk	AD1	Conilon	Group 3	−1.759	1.8	0.641	2	31	16.5
% husk	LB33	Robusta	Group 4	−1.277	1.2	0.324	8	25	16.5
% husk	VR3	Robusta	Group 3	−1.259	1.1	0.296	9	24	16.5
% husk	G30	Robusta	Group 3	−1.397	1.7	0.386	6	30	18.0
% husk	Clementino	Conilon	Group 1	0.146	0.4	0.000	25	11	18.0
% husk	R22	Robusta	Group 3	−1.744	2.1	0.741	4	35	19.5
% husk	VR5	Robusta	Group 1	−0.035	0.7	0.000	21	18	19.5
% husk	R08	Robusta	Group 1	0.190	0.4	0.000	27	12	19.5
% husk	AS1	Robusta	Group 4	−1.200	1.9	0.189	10	32	21.0
% husk	110	Robusta	Group 1	−0.615	1.3	0.012	14	28	21.0
% husk	29	Robusta	Group 1	−0.153	0.9	0.000	20	23	21.5
% husk	AS5	Robusta	Group 3	−1.754	3.1	0.526	3	41	22.0
% husk	RG2	Robusta	Group 3	−1.656	2.9	0.458	5	39	22.0
% husk	JC221	Robusta	Group 5	−2.957	5.1	0.967	1	44	22.5
% husk	LB15	Robusta	Group 3	−1.324	2.5	0.214	7	38	22.5
% husk	VR2	Robusta	Group 3	−1.057	2.1	0.081	11	34	22.5
% husk	A1	Robusta	Group 1	0.184	0.8	0.000	26	20	23.0
% husk	L80	Conilon	Group 1	0.251	0.7	0.000	28	19	23.5
% husk	AS6	Robusta	Group 1	0.503	0.6	0.000	33	16	24.5
% husk	AS2	Robusta	Group 1	0.818	0.5	0.000	37	14	25.5
% husk	VR1	Robusta	Group 1	0.595	0.6	0.000	34	17	25.5
% husk	B11	Robusta	Group 1	0.851	0.5	0.000	38	15	26.5
% husk	B12	Robusta	Group 1	0.949	0.8	0.000	39	21	30.0
% husk	G4	Robusta	Group 1	0.987	0.8	0.000	40	22	31.0
% husk	101	Robusta	Group 1	1.077	1.2	0.000	41	27	34.0
% husk	Pé de Ouro	Robusta	Group 2	1.225	1.2	0.000	42	26	34.0
% husk	07	Robusta	Group 1	0.659	2.4	0.000	35	37	36.0
% husk	RMD	Robusta	Group 1	1.299	1.5	0.000	43	29	36.0
% husk	41	Robusta	Group 1	0.330	4.1	0.000	30	43	36.5
% husk	180	Robusta	Group 1	0.383	6.1	0.000	31	45	38.0
% husk	Neguim	Robusta	Group 1	1.404	2.0	0.000	44	33	38.5
% husk	PDP	Robusta	Group 1	0.447	12.8	0.000	32	48	40.0
% husk	06	Robusta	Group 2	1.974	2.2	0.000	45	36	40.5
% husk	010	Robusta	Group 1	0.745	12.0	0.000	36	47	41.5
% husk	CM1	Conilon	Group 2	2.134	2.9	0.000	46	40	43.0
% husk	G2	Robusta	Group 2	2.464	3.9	0.000	48	42	45.0
% husk	03	Robusta	Group 2	2.253	10.2	0.000	47	46	46.5
FVol/bag	RMD	Robusta	Group 1	−0.939	0.1	0.038	19	2	10.5
FVol/bag	25	Robusta	Group 1	−1.385	0.4	0.052	17	5	11.0
FVol/bag	L80	Conilon	Group 1	−2.137	0.6	0.086	13	12	12.5
FVol/bag	VR2	Robusta	Group 3	−1.886	0.6	0.082	15	11	13.0
FVol/bag	R22	Robusta	Group 3	−1.907	0.6	0.092	14	13	13.5
FVol/bag	AS6	Robusta	Group 1	0.167	0.0	0.014	27	1	14.0
FVol/bag	G30	Robusta	Group 3	−2.514	0.9	0.100	10	20	15.0

Continued on next page

Table S1 continued

Trait	Genotype	Bot.	Div. grp	BLUP	Wricke	Pr(sup.)	Perf.	Stab.	Comp.
FVol/bag	K61	Conilon	Group 1	−3.320	1.5	0.146	7	27	17.0
FVol/bag	AS1	Robusta	Group 4	−0.048	0.5	0.014	25	9	17.0
FVol/bag	B12	Robusta	Group 1	0.588	0.3	0.009	30	4	17.0
FVol/bag	Pirata	Conilon	Group 1	−2.304	1.3	0.074	12	23	17.5
FVol/bag	G1	Robusta	Group 4	1.146	0.2	0.004	32	3	17.5
FVol/bag	LB15	Robusta	Group 3	−4.238	1.6	0.234	6	30	18.0
FVol/bag	B11	Robusta	Group 1	0.219	0.4	0.009	29	7	18.0
FVol/bag	G4	Robusta	Group 1	−0.198	0.8	0.019	22	16	19.0
FVol/bag	VR11	Robusta	Group 1	−0.178	0.7	0.018	23	15	19.0
FVol/bag	AS12	Robusta	Group 1	−0.268	0.8	0.015	21	17	19.0
FVol/bag	Peneirão	Conilon	Group 6	−5.360	2.2	0.305	2	37	19.5
FVol/bag	AD1	Conilon	Group 3	−5.020	2.0	0.286	3	36	19.5
FVol/bag	VR3	Robusta	Group 3	−3.005	1.6	0.154	8	31	19.5
FVol/bag	VR5	Robusta	Group 1	0.845	0.5	0.005	31	8	19.5
FVol/bag	RG2	Robusta	Group 3	−4.278	2.0	0.248	5	35	20.0
FVol/bag	A1	Robusta	Group 1	−2.781	1.8	0.112	9	32	20.5
FVol/bag	101	Robusta	Group 1	1.993	0.4	0.002	35	6	20.5
FVol/bag	JC221	Robusta	Group 5	−1.865	1.4	0.078	16	26	21.0
FVol/bag	Z21	Conilon	Group 6	−8.641	5.2	0.631	1	43	22.0
FVol/bag	29	Robusta	Group 1	0.140	0.8	0.011	26	18	22.0
FVol/bag	AS5	Robusta	Group 3	−4.288	3.7	0.170	4	42	23.0
FVol/bag	41	Robusta	Group 1	2.299	0.6	0.002	36	10	23.0
FVol/bag	R08	Robusta	Group 1	0.199	0.9	0.010	28	19	23.5
FVol/bag	Clementino	Conilon	Group 1	−0.919	1.5	0.032	20	28	24.0
FVol/bag	AS2	Robusta	Group 1	1.507	0.7	0.004	34	14	24.0
FVol/bag	110	Robusta	Group 1	−2.408	2.3	0.081	11	38	24.5
FVol/bag	010	Robusta	Group 1	−0.105	1.6	0.014	24	29	26.5
FVol/bag	LB33	Robusta	Group 4	−1.026	2.6	0.035	18	39	28.5
FVol/bag	LB80	Robusta	Group 1	2.938	1.4	0.001	38	25	31.5
FVol/bag	VR1	Robusta	Group 1	2.947	1.1	0.000	41	22	31.5
FVol/bag	VR4	Robusta	Group 1	3.184	0.9	0.000	42	21	31.5
FVol/bag	06	Robusta	Group 2	3.874	1.4	0.000	44	24	34.0
FVol/bag	G20	Robusta	Group 4	2.345	1.8	0.001	37	33	35.0
FVol/bag	Neguim	Robusta	Group 1	1.355	2.7	0.002	33	40	36.5
FVol/bag	G2	Robusta	Group 2	4.190	1.9	0.000	45	34	39.5
FVol/bag	07	Robusta	Group 1	2.944	2.8	0.000	40	41	40.5
FVol/bag	180	Robusta	Group 1	2.944	7.7	0.000	39	46	42.5
FVol/bag	CM1	Conilon	Group 2	8.679	5.2	0.000	47	44	45.5
FVol/bag	PDP	Robusta	Group 1	3.296	12.1	0.000	43	48	45.5
FVol/bag	03	Robusta	Group 2	5.968	10.6	0.000	46	47	46.5
FVol/bag	Pé de Ouro	Robusta	Group 2	9.877	7.4	0.000	48	45	46.5
FWM/GW	JC221	Robusta	Group 5	−0.021	0.2	0.012	17	6	11.5
FWM/GW	LB80	Robusta	Group 1	−0.014	0.2	0.005	19	5	12.0
FWM/GW	A1	Robusta	Group 1	−0.009	0.1	0.003	22	2	12.0
FWM/GW	R22	Robusta	Group 3	−0.053	0.8	0.133	10	15	12.5
FWM/GW	VR5	Robusta	Group 1	−0.007	0.1	0.002	24	1	12.5
FWM/GW	L80	Conilon	Group 1	−0.027	0.4	0.016	16	12	14.0
FWM/GW	G30	Robusta	Group 3	−0.069	1.2	0.260	6	23	14.5
FWM/GW	VR3	Robusta	Group 3	−0.061	1.0	0.200	9	20	14.5
FWM/GW	Clementino	Conilon	Group 1	−0.042	0.8	0.047	13	16	14.5
FWM/GW	25	Robusta	Group 1	−0.029	0.8	0.014	15	14	14.5
FWM/GW	B12	Robusta	Group 1	0.007	0.1	0.000	28	3	15.5
FWM/GW	K61	Conilon	Group 1	−0.045	1.0	0.070	12	21	16.5
FWM/GW	RMD	Robusta	Group 1	0.008	0.2	0.000	29	4	16.5

Continued on next page

Table S1 continued

Trait	Genotype	Bot.	Div. grp	BLUP	Wricke	Pr(sup.)	Perf.	Stab.	Comp.
FWM/GW	RG2	Robusta	Group 3	−0.068	1.4	0.261	7	27	17.0
FWM/GW	Pirata	Conilon	Group 1	−0.064	1.3	0.143	8	26	17.0
FWM/GW	LB15	Robusta	Group 3	−0.080	2.0	0.351	4	33	18.5
FWM/GW	VR11	Robusta	Group 1	−0.020	1.0	0.007	18	19	18.5
FWM/GW	AD1	Conilon	Group 3	−0.096	2.5	0.417	3	36	19.5
FWM/GW	VR2	Robusta	Group 3	−0.048	1.6	0.056	11	30	20.5
FWM/GW	VR4	Robusta	Group 1	0.026	0.3	0.000	33	8	20.5
FWM/GW	Z21	Conilon	Group 6	−0.128	3.5	0.846	1	41	21.0
FWM/GW	41	Robusta	Group 1	0.027	0.3	0.000	35	7	21.0
FWM/GW	B11	Robusta	Group 1	0.022	0.4	0.000	31	11	21.0
FWM/GW	G4	Robusta	Group 1	0.024	0.4	0.000	32	10	21.0
FWM/GW	Peneirão	Conilon	Group 6	−0.120	4.0	0.517	2	42	22.0
FWM/GW	AS5	Robusta	Group 3	−0.074	2.9	0.116	5	39	22.0
FWM/GW	AS1	Robusta	Group 4	0.006	0.9	0.000	27	17	22.0
FWM/GW	110	Robusta	Group 1	−0.034	1.6	0.014	14	31	22.5
FWM/GW	G1	Robusta	Group 4	0.030	0.3	0.000	36	9	22.5
FWM/GW	AS12	Robusta	Group 1	−0.007	1.2	0.000	23	24	23.5
FWM/GW	R08	Robusta	Group 1	−0.002	1.3	0.000	26	25	25.5
FWM/GW	07	Robusta	Group 1	−0.013	1.6	0.001	20	32	26.0
FWM/GW	AS2	Robusta	Group 1	0.049	0.7	0.000	39	13	26.0
FWM/GW	29	Robusta	Group 1	−0.005	1.6	0.000	25	29	27.0
FWM/GW	VR1	Robusta	Group 1	0.039	0.9	0.000	37	18	27.5
FWM/GW	AS6	Robusta	Group 1	0.019	1.5	0.000	30	28	29.0
FWM/GW	LB33	Robusta	Group 4	−0.010	2.6	0.000	21	38	29.5
FWM/GW	101	Robusta	Group 1	0.058	1.1	0.000	42	22	32.0
FWM/GW	G20	Robusta	Group 4	0.047	2.2	0.000	38	34	36.0
FWM/GW	Neguim	Robusta	Group 1	0.052	2.3	0.000	41	35	38.0
FWM/GW	010	Robusta	Group 1	0.027	6.8	0.000	34	45	39.5
FWM/GW	G2	Robusta	Group 2	0.093	2.5	0.000	44	37	40.5
FWM/GW	06	Robusta	Group 2	0.122	3.2	0.000	46	40	43.0
FWM/GW	180	Robusta	Group 1	0.051	6.9	0.000	40	46	43.0
FWM/GW	PDP	Robusta	Group 1	0.060	9.0	0.000	43	47	45.0
FWM/GW	CM1	Conilon	Group 2	0.137	4.3	0.000	48	43	45.5
FWM/GW	Pé de Ouro	Robusta	Group 2	0.129	4.6	0.000	47	44	45.5
FWM/GW	03	Robusta	Group 2	0.113	14.8	0.000	45	48	46.5
FWM/bag	A1	Robusta	Group 1	−0.408	0.1	0.003	21	2	11.5
FWM/bag	JC221	Robusta	Group 5	−0.833	0.2	0.012	18	6	12.0
FWM/bag	LB80	Robusta	Group 1	−0.648	0.2	0.007	19	5	12.0
FWM/bag	VR5	Robusta	Group 1	−0.265	0.1	0.002	23	1	12.0
FWM/bag	R22	Robusta	Group 3	−2.533	0.8	0.120	10	16	13.0
FWM/bag	L80	Conilon	Group 1	−1.333	0.4	0.015	15	11	13.0
FWM/bag	Clementino	Conilon	Group 1	−1.774	0.8	0.048	13	15	14.0
FWM/bag	25	Robusta	Group 1	−1.332	0.8	0.015	16	13	14.5
FWM/bag	G30	Robusta	Group 3	−3.314	1.2	0.260	7	24	15.5
FWM/bag	VR3	Robusta	Group 3	−2.982	1.1	0.205	9	22	15.5
FWM/bag	B12	Robusta	Group 1	0.260	0.1	0.000	28	3	15.5
FWM/bag	K61	Conilon	Group 1	−2.224	1.0	0.073	12	20	16.0
FWM/bag	RMD	Robusta	Group 1	0.448	0.2	0.000	29	4	16.5
FWM/bag	RG2	Robusta	Group 3	−3.318	1.5	0.258	6	28	17.0
FWM/bag	Pirata	Conilon	Group 1	−3.096	1.4	0.151	8	26	17.0
FWM/bag	VR11	Robusta	Group 1	−1.006	0.9	0.007	17	19	18.0
FWM/bag	LB15	Robusta	Group 3	−3.925	2.1	0.341	4	33	18.5
FWM/bag	41	Robusta	Group 1	1.230	0.3	0.000	33	7	20.0
FWM/bag	AD1	Conilon	Group 3	−4.855	2.6	0.412	3	38	20.5

Continued on next page

Table S1 continued

**Trait**	**Genotype**	**Bot.**	**Div. grp**	**BLUP**	**Wricke**	**Pr(sup.)**	**Perf.**	**Stab.**	**Comp.**
**FWM/bag**	**Z21**	**Conilon**	**Group 6**	**−6.424**	**3.6**	**0.846**	**1**	**41**	**21.0**
FWM/bag	VR4	Robusta	Group 1	1.258	0.3	0.000	34	8	21.0
FWM/bag	B11	Robusta	Group 1	1.111	0.4	0.000	32	10	21.0
FWM/bag	VR2	Robusta	Group 3	−2.417	1.6	0.059	11	32	21.5
FWM/bag	G4	Robusta	Group 1	1.069	0.4	0.000	31	12	21.5
**FWM/bag**	**Peneirão**	**Conilon**	**Group 6**	**−6.229**	**4.1**	**0.521**	**2**	**42**	**22.0**
FWM/bag	AS5	Robusta	Group 3	−3.658	2.8	0.119	5	39	22.0
FWM/bag	AS1	Robusta	Group 4	0.219	0.8	0.000	27	17	22.0
FWM/bag	110	Robusta	Group 1	−1.668	1.6	0.017	14	31	22.5
FWM/bag	G1	Robusta	Group 4	1.378	0.4	0.000	36	9	22.5
FWM/bag	AS12	Robusta	Group 1	−0.265	1.2	0.000	24	23	23.5
FWM/bag	07	Robusta	Group 1	−0.545	1.5	0.001	20	29	24.5
FWM/bag	R08	Robusta	Group 1	−0.002	1.3	0.001	26	25	25.5
FWM/bag	AS2	Robusta	Group 1	2.367	0.8	0.000	39	14	26.5
FWM/bag	29	Robusta	Group 1	−0.236	1.5	0.001	25	30	27.5
FWM/bag	VR1	Robusta	Group 1	1.950	0.9	0.000	37	18	27.5
FWM/bag	AS6	Robusta	Group 1	0.798	1.5	0.000	30	27	28.5
FWM/bag	LB33	Robusta	Group 4	−0.333	2.5	0.000	22	37	29.5
FWM/bag	101	Robusta	Group 1	2.758	1.1	0.000	42	21	31.5
FWM/bag	G20	Robusta	Group 4	2.345	2.3	0.000	38	35	36.5
FWM/bag	Neguim	Robusta	Group 1	2.518	2.2	0.000	40	34	37.0
FWM/bag	010	Robusta	Group 1	1.374	6.5	0.000	35	45	40.0
FWM/bag	G2	Robusta	Group 2	4.736	2.5	0.000	44	36	40.0
FWM/bag	06	Robusta	Group 2	6.079	3.4	0.000	46	40	43.0
FWM/bag	180	Robusta	Group 1	2.652	6.7	0.000	41	46	43.5
FWM/bag	PDP	Robusta	Group 1	2.990	8.7	0.000	43	47	45.0
FWM/bag	CM1	Conilon	Group 2	7.242	4.5	0.000	48	43	45.5
FWM/bag	Pé de Ouro	Robusta	Group 2	6.676	4.7	0.000	47	44	45.5
FWM/bag	03	Robusta	Group 2	5.726	14.5	0.000	45	48	46.5
